# Intracellular alpha-fetoprotein interferes with all-trans retinoic acid induced ATG7 expression and autophagy in hepatocellular carcinoma cells

**DOI:** 10.1038/s41598-021-81678-7

**Published:** 2021-01-25

**Authors:** Shanshan Wang, Rilu Feng, Ying Shi, Dexi Chen, Honglei Weng, Huiguo Ding, Chenguang Zhang

**Affiliations:** 1 Beijing Institute of Hepatology, Beijing You’ An Hospital, Capital Medical University, Beijing, 100069 China; 2 Department of Medicine II, Medical Faculty Mannheim, Heidelberg University, 68167 Mannheim, Germany; 3 Department of Gastroenterology and Hepatology, Beijing You’An Hospital, Capital Medical University, Beijing, 100069 China; 4 Department of Biochemistry and Molecular Biology, School of Basic Medical Sciences, Capital Medical University, Beijing, 100069 China

**Keywords:** Biochemistry, Cancer, Cell biology, Gastroenterology, Oncology

## Abstract

Retinoic acid and retinoid acid receptor (RA-RAR) signaling exhibits suppressive functions in the progression of hepatocellular carcinoma (HCC) through multiple mechanisms. However, whether RA-RAR signaling induces autophagy that contributes its anti-tumor activity in HCC remains elusive. In the current study, the effects of RA-RAR pathway on autophagy were investigated in two HCC cell lines: alpha-fetoprotein (AFP) positive PLC/PRF/5 and AFP negative HLE cells. Cell autophagy was analyzed with western blot for detection of LC3 conversion and p62/SQSTM1 degradation while autophagy flux was assayed using the mRFP-GFP-LC3 reporter. Cell apoptosis and viability were analyzed by caspase-3 activity, TdT-mediated dUTP nick end labeling (TUNEL) assay, and Cell Counting Kit (CCK)-8, respectively. Chromatin immunoprecipitation (ChIP) was employed to detect the binding of RAR onto the promoter of autophagy-relevant 7 (ATG7), and co-immunoprecipitation (CoIP) was used to analyze the interaction of AFP and RAR. The results showed that ATRA dosage and time-dependently induced high levels of cell autophagy in both the PLC/PRF/5 and HLE cells, which was accompanied with up-regulation of ATG7. ChIP assay showed that RAR was able to bind to its responsive elements on ATG7 promoter. Impairment of ATG7 induction or blockade of autophagy with chloroquine aggravated ATRA induced apoptosis of HCC cells. Furthermore, intracellular AFP was able to complex with RAR in PLC/PRF/5 cells. Knockdown of AFP in PLC/PRF/5 cells augmented the up-regulation of ATG7 by ATRA while overexpression of AFP in HLE cells attenuated ATRA induced ATG7 expression and autophagy. Thus, ATRA induced ATG7 and autophagy participated in its cytotoxicity on HCC cells and AFP interfere with the induction of ATG7 and autophagy through forming complex with RAR.

## Introduction

Alpha-fetoprotein (AFP) is the most widely used biomarker for clinical diagnosis of HCC, the fifth most common malignancy and the third leading cause of cancer related death worldwide^1^. Dynamics of serum AFP closely related to the development of the tumor^2^. Besides its clinical utilization as a biomarker, growing evidence has revealed that intracellular AFP functioned as an important signaling molecule, through interaction with a list of proteins, to promote the progression of HCC^3–5^. A series work by us showed that AFP could perturb RA-RAR signaling through interaction with RARα, resulting in transcriptional dysregulation of RAR targets, like *survivin*^3^, *Fn14*^4^, *GADD45*^5^, *GADD153*^6^ etc. in HCC cells. ATRA or its chemical derivatives have long been tested as candidates for treatment of HCC as single reagent or in combination with other clinically used drugs^7,8^, the results was, however, far to be satisfactory, which could be partially attributed to the perturbation of AFP and the resultant dysregulation of RAR target genes. Given the profound effect of ATRA on HCC cells and the broad distribution of retinoic acid response elements (RAREs) in human genome, whether other target genes of RA-RAR signaling and related biological process is regulated by AFP in HCC cells is tempting to investigation.

Macroautophagy (referred to as autophagy hereafter) is a conserved degradation system for damaged, misfolded, or senescent cellular components, like organelles or certain proteins to maintain cellular homeostasis^9^. About 40 autophagy related genes (ATGs) have been identified to date and participate in the whole process of autophagy that was mainly composed of initiation and elongation of the phagophore, autophagosome formation, autophagosome fusion with lysosomes and final degradation of the intracapsular products, in a highly ordered manner^10^. Important signaling molecules like AMPK, mTOR, PI3K/Akt etc. showed potent regulation on autophagy^11^. For example, mTORC1 inhibited autophagosome formation elicited by ULK1 (ATG1) while activated AMPK was able to inhibit mTORC1and directly phosphorylates ULK1, leading to autophagy initiation^12^. RA-RAR signaling has also been implicated in the modulation of autophagy through multiple mechanisms in different cell types. In acute promyelocytic leukemia (APL) cells, ATRA was able to induce autophagy through inhibition of mTOR pathway, which contributed to the degradation of the fusion oncoprotein PML/RARα, resulting in cell differentiation and the remission of the tumor^13,14^. In breast cancer cells, ATRA was reported to induce autophagy dependent on RARα, and ablation of autophagy promoted ATRA induced apoptosis of the cancer cells^15^. Fang et al. also suggested induction of autophagy and expression of a panel of ATGs by ATRA in Hepa1-6 mouse hepatoma cells^16^, however, the generality of autophagy induction by ATRA in HCC and the underlying mechanism remains to be further addressed.

Conventional chemotherapeutic drugs for HCC like doxorubicin, oxaliplatin, cisplatin have all been reported to induce autophagy in vitro and in vivo that seemed be protective for the cells under treatment, for inhibition of autophagy was able to enhance the anti-tumor activity of these drugs^17^. Multiple ATGs and related signaling pathways were shown to regulate sensitivity of HCC cells to chemo- or targeted reagents, which might hold potential therapeutic potentials^18^. We recently provided intriguing evidence that AFP played a suppressive role in the maintenance of the basal level of autophagy in HCC cells through interaction with PTEN, which led to inhibition of its phosphatase activity and subsequent over-activation of PI3K/Akt/mTOR, and finally promoted cell survival^19^. As AFP also interacted with RARα and perturbed RA-RAR signaling as well as the anti-tumor effect of ATRA in HCC cells, whether this perturbation also participates in regulation of cell autophagy is of great interest to be investigated.

In the present study, we found that ATRA robustly induced autophagy and transcriptional up-regulation of ATG7 in human HCC cells, which played protective roles for ATRA treated cells. Furthermore, AFP interacted with RARα and attenuated its regulation on ATG7 expression and autophagy. Our results were supposed to be helpful for developing novel therapeutics for HCC composed of ATRA and autophagy inhibition reagents, where the level of AFP needs to be taken into consideration.

## Results

### ATRA induced autophagy in PLC/PRF/5 and HLE cells

**Figure 1:**
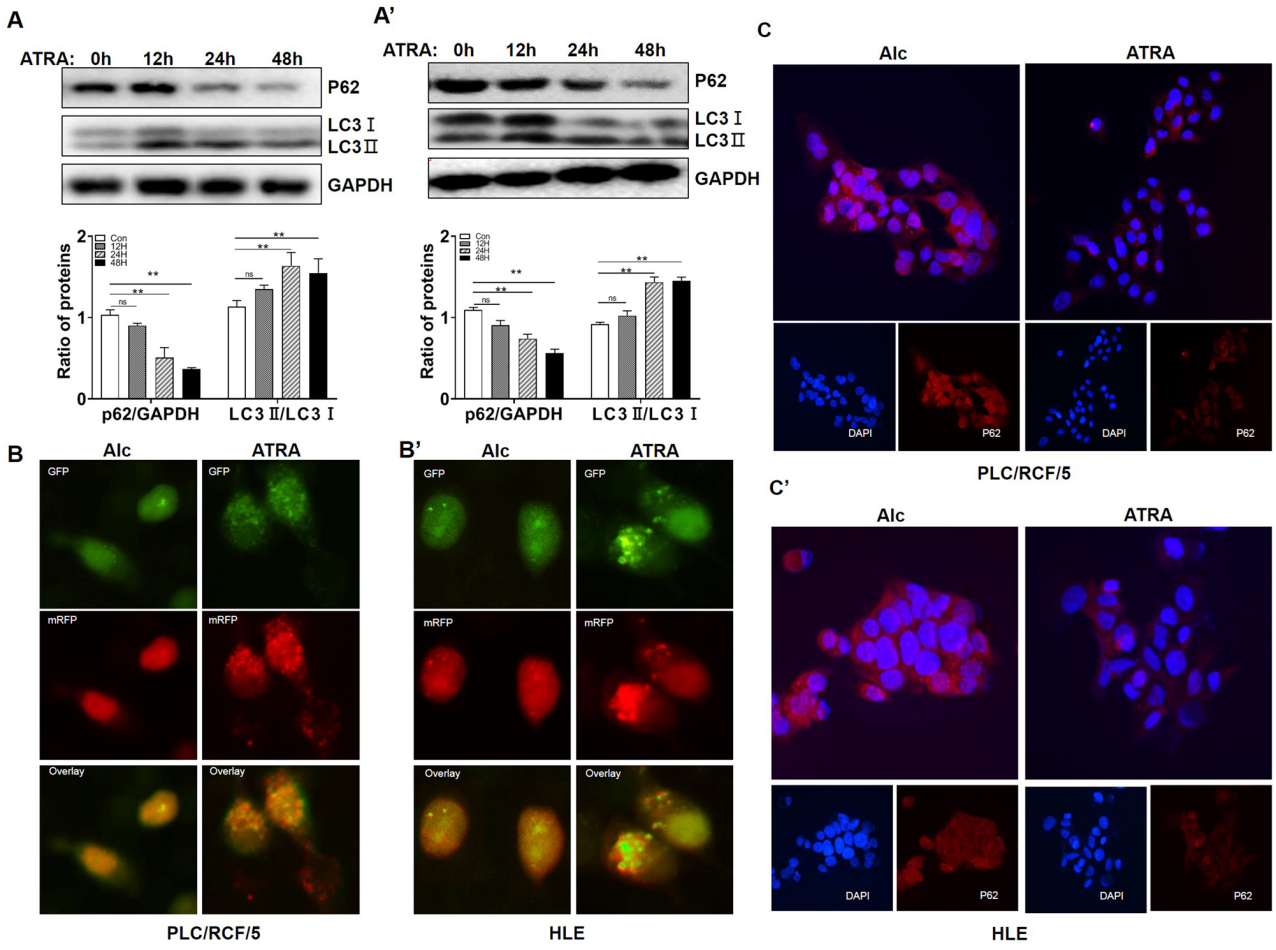
ATRA promoted autophagy in PLC/PRF/5 and HLE cells. A. Western blotting analyses of the level of p62/SQSTM1 and LC3 in PLC/PRF/5 (**A**) and HLE (**A’**) cells upon ATRA treatment at indicated time points (Top). GAPDH was used as loading control. Densitometry of the blots were quantified, and the ratio of p62/SQSTM1 to GAPDH, or LC3II to LC3I were calculated (Bottom). **P < 0.01, one-way ANOVA. ns: no significance. B. PLC/PRF/5 (**B**) and HLE (**B’**) cells were transduced with tandem mRFP-GFP-LC3 adenovirus and were then subjected to ATRA for 24 h. Representative images of fluorescent LC3 puncta are shown. (**C**) and (**C’**). The expression of p62/SQSTM1 was detected with immunofluorescence and was observed under the fluorescence microscopy. Nuclei were stained with DAPI (blue). p62/SQSTM1 were labeled with TRITC (red). All the shown images are representative of three independent experiments. Ethyl Alcohol (Alc) was used as a solvent and as a control.

To investigate if ATRA was able to induce autophagy in HCC cells, HCC cells were treated with 40 μM ATRA, and Ethyl Alcohol (Alc), the solvent of ATRA, was used as negative control. As shown in Fig. 1A,A’, ATRA treatment significantly promoted cell autophagy in a time dependent manner, in both PLC/PRF/5 and HLE cells, as demonstrated by up-regulation of LC3-II and decrement of p62/SQSTM1 at the protein level. Chloroquine, an inhibitor of autophagy, blocked ATRA induced LC3 conversion and p62/SQSTM1 degradation (supplementary Figure 3A and B), supporting the induction of autophagic flux by ATRA in HCC. To further evaluate the autophagic flux in ATRA treated HCC cells, we employed the mRFP-GFP-LC3 adenovirus vectors. PLC/PRF/5 and HLE cells transfected with mRFP-GFP-LC3 adenovirus were added with 40 μM ATRA and cultured for 24 h. Numbers of GFP and mRFP dots per cell were both significantly increased under ATRA treatment (Fig. 1B,B’, and quantification in supplementary Figure 1C). Immunofluorescence analyses further confirmed that ATRA significantly reduced the level of p62/SQSTM1 in both PLC/PRF/5 and HLE cells (Fig. 1C,C’). All these results indicated that ATRA induced autophagy in HCC cells. The activation of RA-RAR signaling in HCC cells was verified with nuclear accumulation of RAR as demonstrated with western blot analyses for nuclear proteins (supplementary Figure 1A) and cellular immunofluorescence (supplementary Figure 1B).

### ATRA-RAR signaling regulated transcription of ATG7

**Figure 2:**
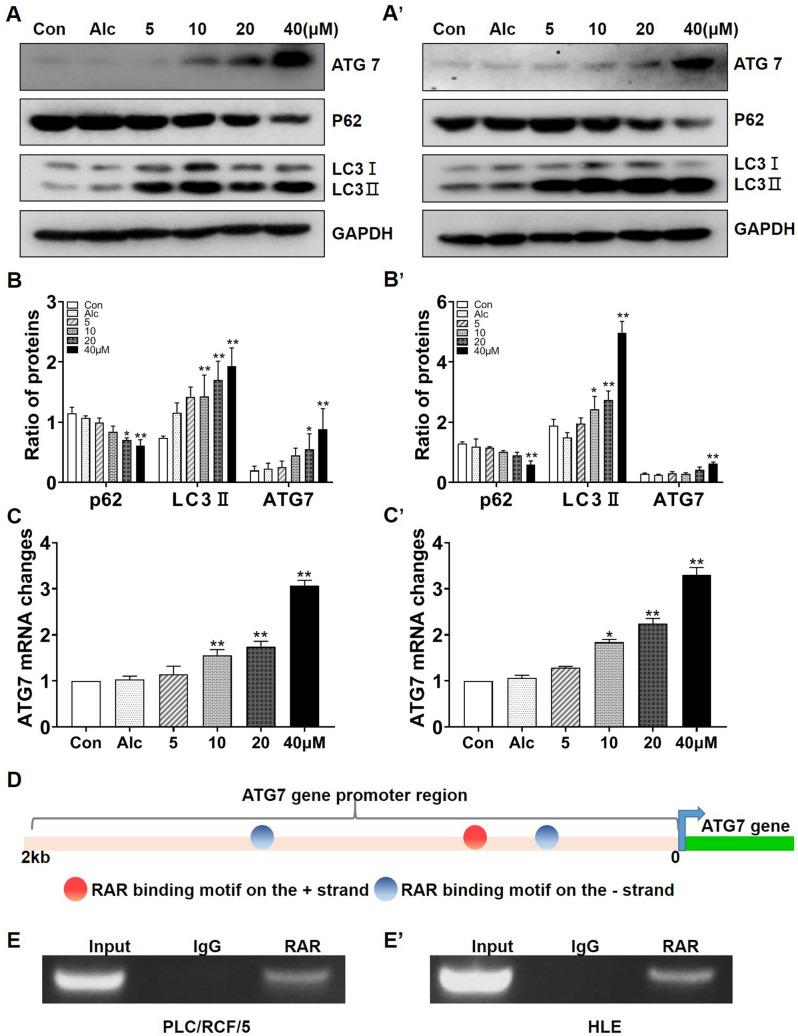
RAR signal cascade regulated transcription of ATG7 in HCC cells. (**A**) and (**B**). Various concentrations of ATRA were used to treat HCC cells for 24 h. Expression of the ATG7 protein, p62/SQSTM1 and LC3 in PLC/PRF/5 (**A**) and HLE (**A’**) cells was detected using Western blotting. GAPDH was used as loading control. (**B**) and (**B’**). Densitometry of the blots in Figure A and A’ were quantified and the ratio of p62/SQSTM1 to GAPDH or LC3II to LC3I were calculated. *P < 0.05, **P < 0.01 compared with control group, one-way ANOVA. (**C**) and (**C’**). The mRNA expression level of ATG7 gene in PLC/PRF/5 (**C**) and HLE (**C’**) cells were analyzed with qRT-qPCR. *P < 0.05, **P < 0.01 compared with control group, one-way ANOVA. (**D**) Schematic overview of the RAR binding sites on ATG7 promoter predicted with JASPAR website (http://jaspar.genereg.net/). (**E**) and (**E’**). ChIP assays for RAR binding onto ATG7 promoter were carried out in PLC/PRF/5 (**E**) and HLE (**E’**) cells.

To further reveal the potential molecular mechanism underlying ATRA induced autophagy in HCC cells. Expressions of ATG5, Beclin1 and ATG7 were evaluated with RT-qPCR in PLC/PRF/5 and HepG2 cells. According to preliminary experimental results, expression of ATG7, an E1-like activating enzyme for autophagosome formation^20^, was most significantly up-regulated under ATRA treatment in both HCC cell lines, while Beclin1 and ATG5 were not (supplementary Figure 2A, B, C). ATG5 even manifest a decrement at the mRNA level, but no similar expression trend was seen at the protein level which needs further investigation (supplementary Figure 2D and E). We thus focused on potential regulation of ATG7 by ATRA-RAR in the following studies. Western blotting and qRT-PCR showed that ATRA induced robust increment of ATG7 in both PLC/PRF/5 (Fig. 2A,B) and HLE (Fig. 2A’,B’) cells, in a dose-dependent manner, reaching maximum at 40 μM. Similar results were observed at the mRNA level with the qRT-qPCR assay (Fig, 2C,C’). The alteration of ATG7 at the mRNA level prompted us to investigate if ATG7 was transcriptionally regulated by RAR. Two adjacent binding sequence for RAR was discovered at the proximal promoter of ATG7 (Fig. 2D). To validate if RAR was able to bind to the region, ChIP assays were performed. As shown in Fig. 2E,2E’, RAR was able to bind to the 5′-flanking regions containing its responsive elements at the ATG7 promoter in both PLC/PRF/5 and HLE cells, indicating a direct transcriptional regulation of ATRA-RAR signaling on ATG7 via RAR.

### ATG7 played a protective role in HCC during ATRA treatment

**Figure 3:**
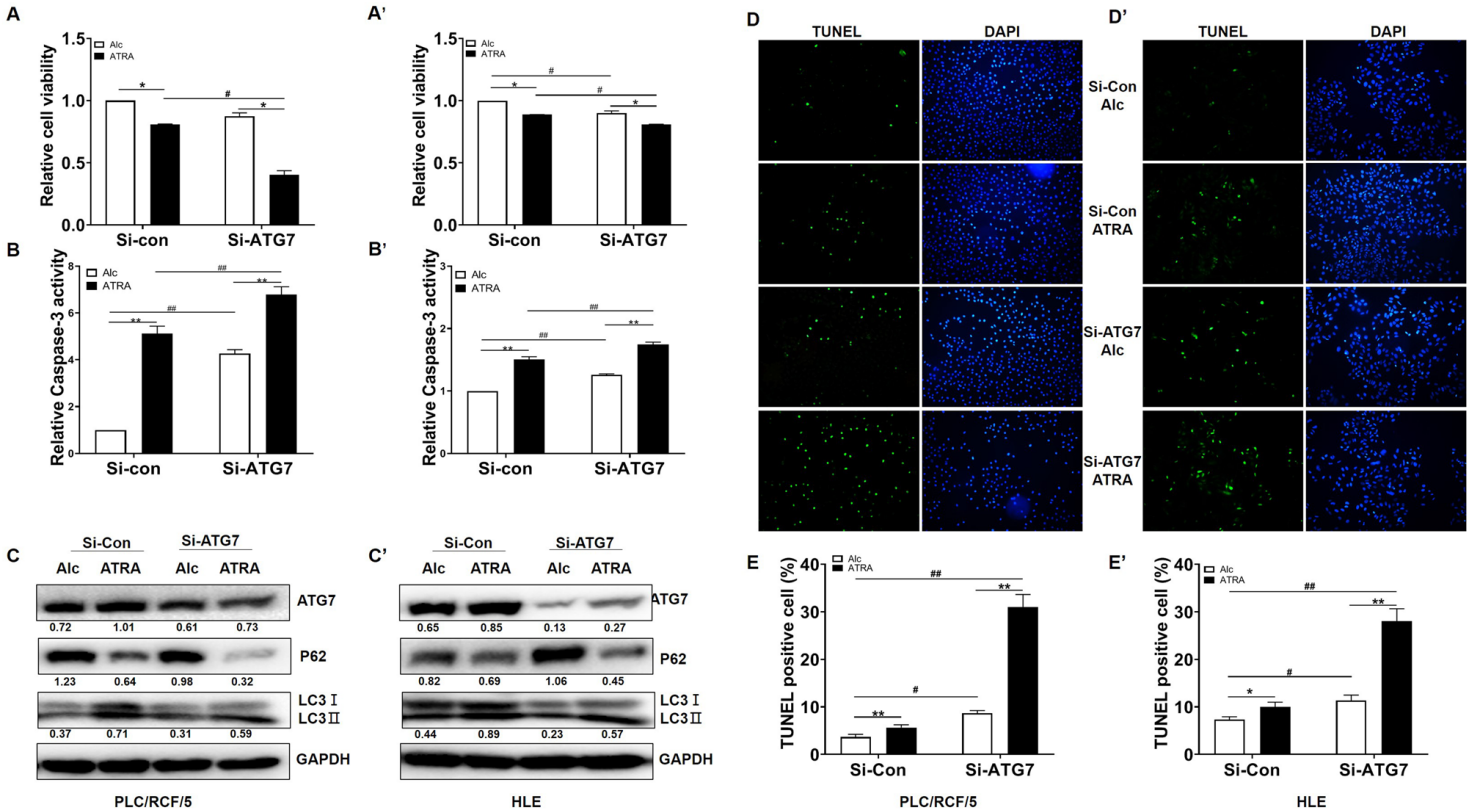
ATG7 prevented ATRA-induced HCC cell apoptosis. (**A**) and (**A’**). PLC/PRF/5 and HLE cells were pre-treated with siRNAs (si-con and si-ATG7) for 24 h, and was then treated with ATRA at final concertation of 40 μM for further 24 h. PLC/PRF/5 cells (**A**) and HLE (**A’**) cell viability was determined by CCK-8 assays. * and ^#^*P* < 0.05. **and ^##^*P* < 0.01, two-way ANOVA. (**B**) and (**B’**), Caspase-3 activity of PLC/PRF/5 cells (**B**) and HLE (**B’**) cells were also detected following treatment as in (**A**) and (**A’**). * and ^#^*P* < 0.05. ** and ^##^*P* < 0.01, two-way ANOVA. (**C**) and (**C’**). Protein level for ATG7, p62/SQSTM1 and LC3 were detected upon ATG7 knockdown and ATRA treatment. GAPDH was used as loading control. Densitometry of the blots were quantified, and the ratio of ATG7 and p62/SQSTM1 to GAPDH, and LC3II to LC3I were calculated and marked under the corresponding lanes. (**D**, **D’**) and (**E**, **E’**). Apoptosis rates of PLC/PRF/5 cells (**D** and **E**) and HLE (**D’** and **E’**) cells were determined with TUNEL assay. * and ^#^*P* < 0.05. ** and ^##^*P* < 0.01, two-way ANOVA.

We next investigated whether the induction of ATG7 expression and autophagy were functional in ATRA treated HCC, or merely indicators for the activity of ATRA. CCK-8 and caspase-3 activity assays were carried out. The CCK-8 results shown that cell viability was further decreased with knockdown of ATG7 in response to ATRA in both PLC/PRF/5 and HLE cells (Fig. 3A, A´), which was accompanied with increment of caspase-3 activity (Fig. 3B,B´). Meanwhile, knockdown of ATG7 mildly reduced basic level of autophagy in HCC cells, as evidenced with P62/SQSTM1 accumulation and reduced LC3 conversion (Fig. 3C,C’, lane 3 versus lane 1). Impairment ATG7 expression also attenuated ATRA induced LC3II conversion. However, degradation of p62/SQSTM1 upon ATRA treatment was further aggregated by ATG7 siRNA (Fig. 3C,C’, lane 4 versus lane 2), suggesting alternative degradation pathway(s) activation with simultaneous ATRA stimulation and ATG7 silence for p62/SQSTM1. The protective role of ATG7 for ATRA treated cells was also validated by TUNEL assay (Fig. 3D,D’,E,E’). Furthermore, inhibition of autophagy with 40 μM chloroquine also resulted in further decrement of cell viability of HCC cells by ATRA (supplementary Figure 3C and D). These results indicated that ATG7 and autophagy, played protective roles in ATRA induced apoptosis of HCC cells.

### AFP interacted with RAR in HCC cells

**Figure 4:**
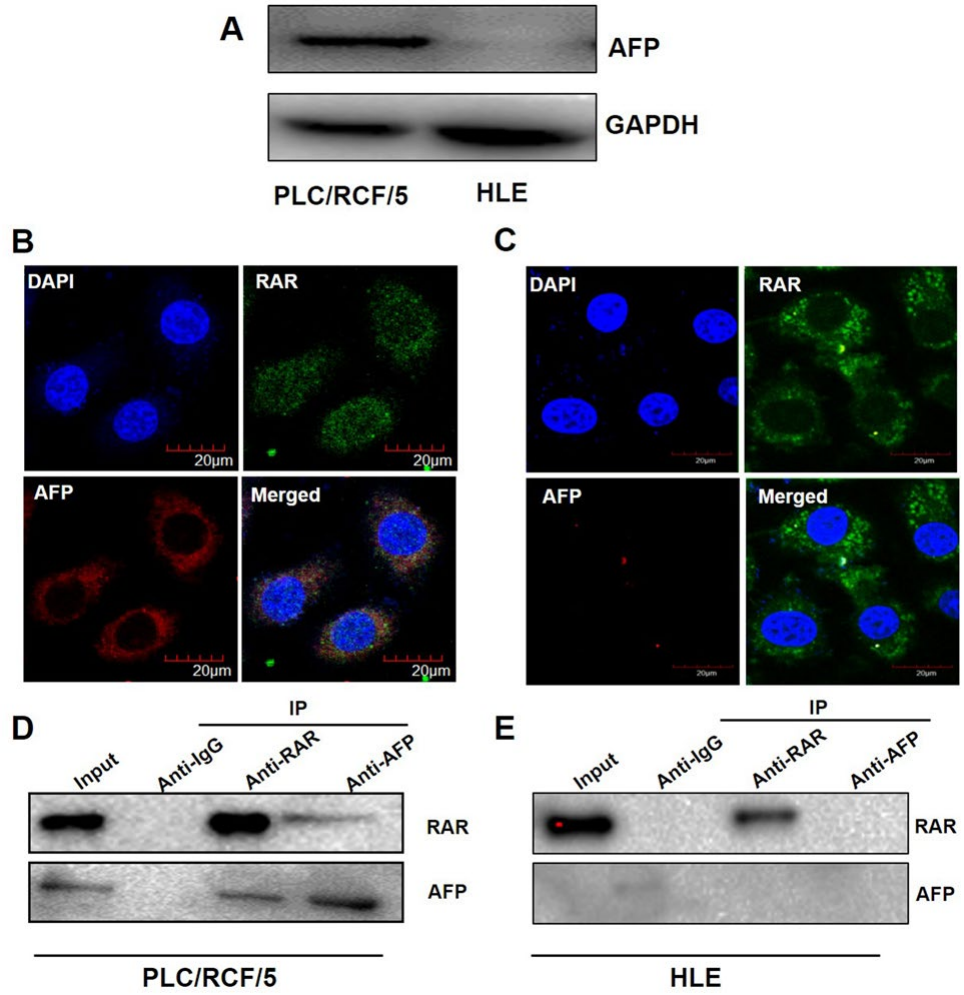
AFP interacted with RAR in cytoplasm of HCC cells. (**A**) Western blotting was used for analysis of expression of AFP in PLC/PRF/5 and HLE cells. (**B**) and (**C**). Expression and localization of AFP and RAR were analyzed with confocal microscopy in PLC/PRF/5 (**B**) and HLE (**C**) cells. (**D**) and (**E**). In PLC/PRF/5 (**D**) and HLE (**E**) cells, Co-IP was employed to detect the interaction of AFP and RAR. The images captured by confocal microscope are representatives of experiments that were repeated at least three times.

To investigate whether AFP could possibly regulate ATRA-RAR mediated autophagy, western blotting analyses were first employed to detect the endogenous expression of AFP in PLC/PRF/5 and HLE cells. As previously reported, AFP protein was undetectable in HLE cells, but robustly expressed in PLC/PRF/5 cells (Fig. 4A). Further analyses with confocal microscopy showed that AFP and RAR co-localized in cytoplasm in PLC/PRF/5 cell (Fig. 4B), but not in HLE cells (Fig. 4C), which were further confirmed by Co-IP analysis (Fig. 4D,E).

### AFP perturbed ATRA induced ATG7 expression in HCC cells

**Figure 5:**
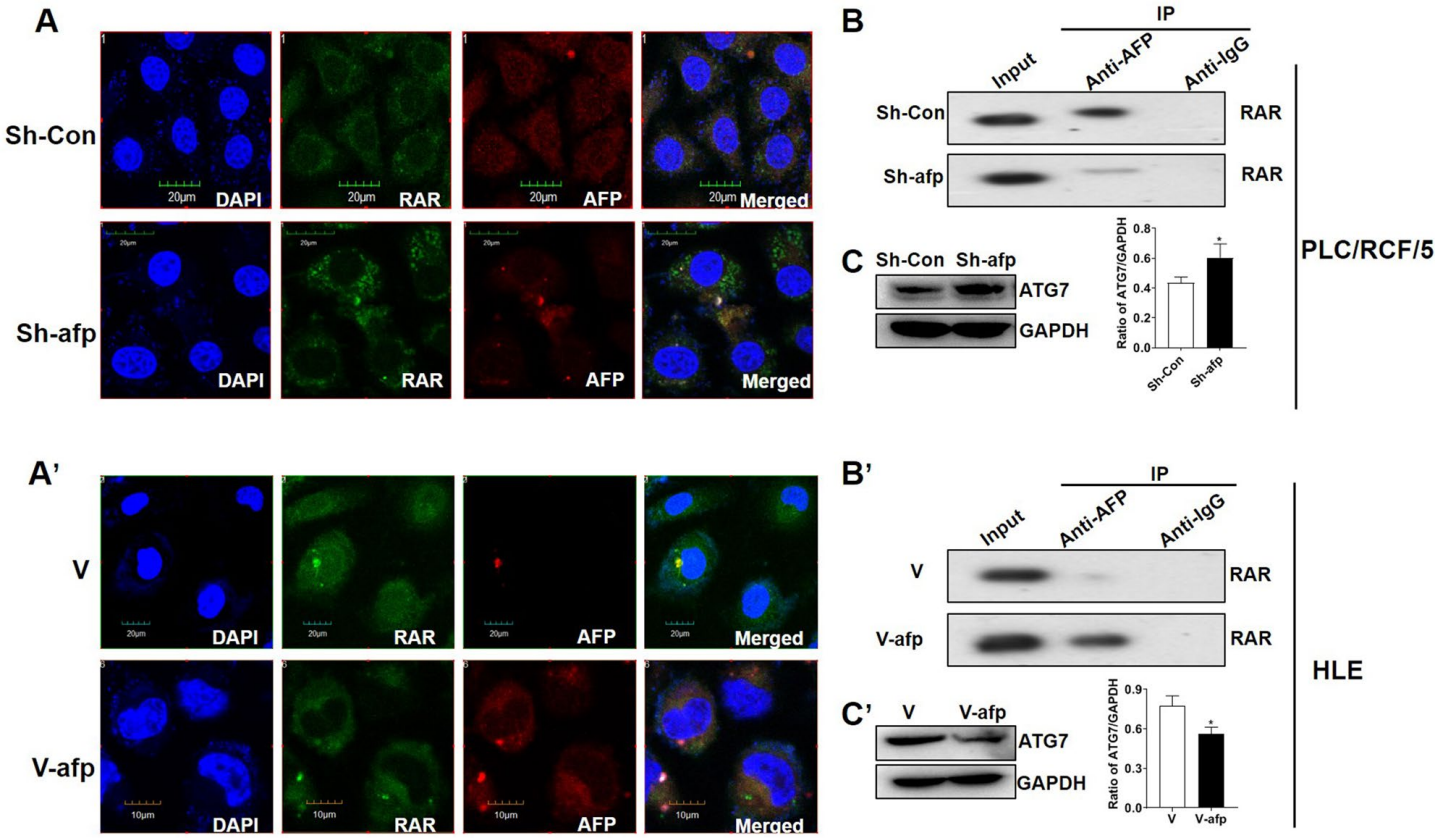
AFP attenuated RAR mediated of ATG7 expression in HCC cells. (**A**) and (**A’**). Localization of AFP (red) and RAR (green) in AFP–shRNA923-transfected PLC/PRF/5 cells (**A**) and pcDNA3.1-afp-transfected HLE cells (**A’**) were detected with immunofluorescence and observed with confocal microscopy. (**B**) and (**B’**). Co-IP was carried out to detect the interaction between AFP and RAR in AFP knockdown or ectopic expression in PLC/PRF/5 cells (**B**) or HLE cells (**B’**), respectively. (**C**) and (**C’**). Effects of AFP knockdown or ectopic expression on ATG7 expression in PLC/PRF/5 cells (**C**) and HLE (**C’**) cells were analyzed by Western blotting. GAPDH was used as loading control. Densitometry quantification were performed, and ratio of ATG7 to GAPDH were calculated. *P < 0.05, Two tailed Student’s *t* test.

To further investigate if AFP was involved in ATRA-RAR mediated ATG7 expression by interacting with RAR, AFP was first knockdown by specific shRNA in PLC/PRF/5 cells. Immunofluorescence and confocal microscopy assays showed that AFP expression was obviously depleted upon specific shRNA transfection compared with scramble shRNA (Fig. 5A). Following AFP depletion, binding of AFP with RAR was significantly decreased as demonstrated by Co-IP assay in PLC/PRF/5 cells (Fig. 5B). On the contrary, when AFP was introduced into HLE cells with pcDNA3.1-afp vectors (Fig. 5A’), notable interaction between AFP and RAR was observed as shown by Co-IP results (Fig. 5B’), accompanied with co-localization of AFP and RAR in the cytoplasm (Fig. 5A´). One intriguing phenomenon was observed that alteration of the intracellular AFP level not only changed its interaction with RAR, but also exhibit a negative regulation on the protein level of RAR itself (Fig. 5A,A’), which needs further investigation.

Interaction between AFP and RAR was able to disrupt the transcriptional regulation of RAR on its targets, we wonder whether it was also the case in ATG7. Not surprisingly, when AFP was down regulated by shRNA in PLC/PRF/5 cells, the ATG7 protein level was remarkably increased compared with the control in untreated conditions (Fig. 5C). On the other hand, AFP expression in HLE cells resulted in an apparent reduction of ATG7 protein (Fig. 5C’). Similar results were also observed under ATRA treatment conditions, although to a less extent in HLE cells (supplementary Figure 4A,B). However, knockdown of AFP in PLC/PRF/5 did not obviously alter the effect of ATRA on p62/SQSTM1 degradation and LC3 conversion while ectopic expression of AFP in HLE cells significantly attenuated ATRA induced alterations of p62/SQSTM1 and LC3II, possibly suggesting dose dependence of AFP on ATRA induced autophagy as well as involvement of other regulators beyond ATG7 in this process (see discussion).

## Discussion

In the present study, ATRA treatment robustly induced autophagy in HCC cells through transcriptional up-regulation of ATG7. Mechanistically, ATRA induced nuclear accumulation of RAR, which bound onto the promoter region of ATG7 that harbors RAR binding motifs. Intracellular AFP interacted with RAR and exhibited an inhibitory effect on nuclear accumulation of RAR, resulting in down-regulation of ATG7 of HCC cells. Functional studies indicated a protective role of the induced expression of ATG7 and autophagy, and impairment of ATG7 induction or blockade of autophagy further aggravated ATRA induced cell apoptosis (supplementary Figure 5).

ATRA has long been used clinically to induce differentiation of APL cells, where the relationship between ATRA and autophagy were mostly studied. An array of ATGs and important regulators of autophagy, including ATG1, ATG5, Beclin1, mTOR, PI3KC3, WIPI and TFEB, DRAM etc., were implicated in ATRA induced autophagy^14,21–24^. In other cell types, including several other solid tumor types, ATRA was also able to induce autophagy^15,25^. In these studies, expression alterations of certain ATGs or signaling molecules were always displayed as the underlying mechanisms, which seemingly was not powerful enough to establish direct links between ATRA and autophagy, as the involvement and the function of RAR always lacked. For example, ATRA induced autophagy in human B cells through mTOR inhibition^26^, and induced autophagy in APL cells via potent up-regulation of TFEB^23^, how the inhibition or promotion occurred, directly through RAR or by other alternative pathways? The present study directly linked ATRA and autophagy in HCC cells with RAR mediated transcriptional activation of ATG7. Of course, as ATRA was able to elicit a number of other downstream signaling pathways^27^, it still cannot rule out the possibility that other regulators were also involved in ATRA induced autophagy in HCC cells.

Autophagy and apoptosis are generally discrete cellular processes mediated by distinct groups of molecules^28^. However, they often occurred in the same cell under stresses, and interactions among molecules involved in apoptosis and autophagy dictated the progression of each process. Autophagy under moderate levels of stress generally exhibited a protective role and inhibited cell apoptosis^29^. For example, blockade of autophagy by knockdown Atg1, Atg5 and PI3KC3 etc. or by specific autophagy inhibitors like 3-methyladenine (3-MA) impaired ATRA induced differentiation of APL cells^14^, suggesting the necessity of autophagy for the primary function of ATRA. In breast cancer cells, autophagy was reported to be cell protective and inhibition of autophagy genetically or pharmacologically resulted in robust apoptosis^15^. In the current study, as in most cases of chemotherapeutic drug treatment, ATRA induced autophagy also played a protective role for the cancer cells, as ATG7 knockdown or CQ treatment both potentiated ATRA induced apoptosis (Fig. 3 and Supplementary Figure 3), though overwhelmed by the potency of ATRA. Besides the general protective role of autophagy, ATG7 was also able to directly bind to p53 to modulate cell survival under metabolic stress independent of its E1-like enzymatic activity^30^. Both mechanisms may function in HCC cells under ATRA treatment. ATRA has been shown to induce differentiation of tumor initiating cells in HCC, and potentiated the cytotoxicity of chemotherapeutic drugs like cisplatin^31^. It has also been shown to enhance the anti-tumor activity of sorafenib through activation of AMPK^32^, a potent regulator for autophagy induction. Together with the reports that most reagents for HCC treatment induced autophagy, and autophagy was cell protective in most cases, it is plausible to consider combinational use of chemotherapeutic drugs with ATRA and autophagy inhibitors like chloroquine to improve the efficacy of chemotherapy for HCC.

We recently reported that AFP was able to block basal level of autophagy in HCC cells through direct sequestration of PTEN, which leads to overactivation of PI3K-Akt-mTOR cascade^19^. Together with current results, AFP was thus able to disrupt both the basal and ATRA induced autophagy through interaction of different partners (PTEN or RAR) and modulation of different autophagy regulators (PI3K/Akt/mTOR or ATG7). However, whether autophagy and AFP played identical roles in these conditions still needs further illustration. Under basal conditions, increased level of autophagy with AFP knockdown was accompanied with PTEN overactivation and increased cell apoptosis^19^. However, to what extent did autophagy contribute to increased apoptosis could not be figured out with the evidence provided; In ATRA treated conditions, ATG7 played cell protective roles and AFP perturbed ATG7 expression. From this point of view, AFP seemed to contribute to ATRA induced cell death, which was obviously against its well-known tumor promoting function. The discrepancy could be explained that PTEN and RAR were both able to elicit multiple important downstream signalings or effectors to exert their potent anti-tumor functions. AFP, as an important oncoprotein for HCC, interacted with both molecules to counteract their biological functions, with autophagy induction being one of them. Interestingly, ATRA has been shown to induce expression of PTEN, which contributed its anticancer activity in APL cells^33^. Whether similar crosstalk exists, and how overexpression of AFP could orchestrate those signaling pathways in HCC required further investigation.

Some discrepancies still exist in the current study. The first one is that impairment of ATG7 expression upon ATRA significantly attenuated LC3 conversion but further promoted p62/SQSTM1 degradation (Fig. 3C,C’). It was probable that blockade of autophagy with ATG7 knockdown enhanced proteotoxicity of ATRA in HCC cells, activating alternative protein degradation pathways like the proteasome or endosomal microautophagy, both of which has been involved in p62/SQSTM1 degradation^34,35^. The second is that the extent of expression alteration for ATG7 was more obvious upon AFP knockdown in PLC/PRF/5 than its ectopic expression in HLE cells while the inhibitory effect of AFP on autophagy was much better manifested in HLE cells (supplementary Figure 4). Explanations for this might include: (1) the dosage alteration of AFP was much more prominent in the ectopic expression model in HLE than in the knockdown model in PLC/PRF/5; (2) other regulators and pathways of autophagy like mTOR were affected by the higher dosage of AFP in HLE cells; (3) further up-regulation of ATG7 upon AFP knockdown participated non-autophagic processes as previous suggested^36^. More detailed investigations are needed to be taken to address these issues.

## Materials and methods

### Cell lines

AFP-producing hepatocellular carcinoma cell line PLC/PRF/5 cells and AFP-non producing cell line HLE were both maintained in a 5% CO_2_ incubator and cultured in DMEM medium supplemented with 10% FCS.

### Western blotting

For western blotting, total cell proteins from each sample were extracted with radioimmunoprecipitation (RIPA, Thermofisher, USA) cell lysis buffer containing protease inhibitor cocktail (CST, USA), 15 μg of which were then subjected to 12% SDS-PAGE. Electrophoretic transfer of proteins from gels onto nitrocellulose membrane was carried out in a transblotting cell. Membranes were blocked by immersing in 5% nonfat milk (w/v) /PBS for 1 h, and then incubated with primary antibodies at 4 °C for overnight. After rinsing with PBS/0.1% Tween-20, membranes were incubated with horseradish peroxidase-conjugated secondary Ab. The signals were visualized by incubation with the Enhanced Chemiluninescence kit and exposure on an X-ray film. Densitometry quantification of the bands were performed with Image J software. Primary and secondary antibodies used in this study are listed in Table 1.

**Table 1:** Antibodies and Sequences of oligonucleotides.

**Primary and sencondary antibodies**		
P62/SQSTM1	Cell Signaling Technology	CST-5114S
LC3	Sigma-Aldrich	L7543-100UL
ATG7	Cell Signaling Technology	CST-8558S
RAR	Santa Cruz Biotechnology	sc773
AFP	Santa Cruz Biotechnology	sc8399
HDAC	Cell Signaling Technology	CST-3949
GAPDH	Cell Signaling Technology	CST-2118S
Goat anti-rabbit IgG-HRP	Santa Cruz Biotechnology	sc-2004
Goat anti-mouse IgG-HRP	Santa Cruz Biotechnology	sc-2354
Goat anti-Rabbit IgG Alexa Fluor 488	Invitrogen	R37116
Goat anti-Mouse IgG Alexa Fluor 594	Invitrogen	R37121
**Oligonucleotides sequences**		
**Primers for RT-qPCR**		
**ATG7**		
Sense	5′-AAGAAATAATGGCGGCAGCT-3'	
Antisense	5′-ACCCAACATCCAAGGCACTA-3'	
**GAPDH**		
Sense	5′-TGAAGGTCGGAGTCAACGGA-3'	
Antisense	5′-CCTGGAAGATGGTGATGGGAT-3'	
**Primers for ChIP-qPCR**		
**ATG7**		
Sense	5′-TTGGGTTGTTGCATTTCTGA-3'	
Antisense	5′-CCATCCTAATTGGCCTGTGT-3'	
**AFP-shRNA**		
Sense	5′-CACCGAACGTGGTCAATGTATAATTCAAGAGATTATACATTGACCACGTTCTTTTTTG-3'	
Antisense	5′-GATCCAAAAAAGAACGTGGTCAATGRATAATCTCTTGAATTATACATTGACCACGTTC-3’	

### Immunofluorescence staining

Cells were seeded on coated glass coverslips, then fixed with 4% paraformaldehyde. After incubated with PBST buffer (containing 0.05% triton-100, 0.5% BSA in PBS) at room temperature for 10 min, the cells were washed with PBS twice followed with blocking using 1% BSA at room temperature for 10 min. The primary antibodies were then added and incubated at 4 °C overnight. After rinsing with PBST for 10 min, secondary antibodies, Alexa Fluor 594 and 488 (Thermofisher, USA) were incubated with cells for one hour in room temperature followed by addition of DAPI for counterstaining of the nuclei. Cells images were captured with a Laser Confocal Microscope (Leica TCS STED-3X, Germany).

### Quantitative real-time reverse transcription PCR (RT-qPCR)

Expression of ATG7 at the mRNA level was evaluated by quantitative real-time reverse transcription PCR (RT-qPCR) assay. Briefly, total RNA was extracted from HCC cells using the RNeasy Mini Kit (Qiagen, Germany) according to the manufacturer’s instructions. cDNA was then synthesized by reverse transcription of the extracted RNA using a SuperScript II First-stand Synthesis System (Invitrogen, USA). SYBR Green was used to detect the dsDNA products during the real-time PCR reaction. The mRNA content was normalized to the housekeeping gene GAPDH and fold change was calculated with the 2^−ΔΔCT^ method. All primer sequences for RT-qPCR are listed in Table 1.

### Co-immunoprecipitation (Co-IP)

Co-IP experiments for evaluation of the interaction between AFP and RAR were performed in PLC/PRF/5 and HLE cells. HCC cells were lysed in RAPI buffer containing 1% protease inhibitor cocktails. The lysates (1 mg/500 μl) were then incubated with 1 μg anti-AFP, anti-IgG and anti-RAR antibodys for overnight at 4 °C, then added 100 μl Protein A SepHarose agarose beads (CL-4B) (17-0780-01, GE, USA) for further incubation for at least 8 h at 4 °C. Collected these beads and washed 3 times with 1 ml PBS contain 1% protease inhibitor cocktails before boiling them in the loading buffer, and then Western blotting analyses were performed.

### Transient transfection

The AFP-expressing plasmid (pcDNA3.1-afp) was used to overexpress AFP in HLE cells, and the AFP interference shRNA (AFP-shRNA923) was employed to knockdown endogenous AFP in PLC/PRF/5 cells. ATG7 siRNA (sc-41447) and control siRNA (sc-37707) were used to silence the ATG7 mRNA in the PLC/RCF/5 and HLE cells. In brief, HLE were transfected with pcDNA3.1-afp or control vector, and PLC/PRF/5 cells were transfected with AFP-shRNA923 or scramble control shRNA with Lipofectamine 3000 (Invitrogen, USA) according to the manufacturer’s instructions. After 24 h of transfection, the expression of AFP and ATG7 were evaluated by Western blotting and the interaction of AFP and RAR was examined by Co-IP. PLC/RCF/5 and HLE cells were transfected with ATG7 siRNA or control siRNA for 24 h, followed with ATRA treatment for another 24 h, and then subjected to functional evaluation.

### Chromatin immunoprecipitation and PCR (ChIP-PCR)

ChIP was performed to verify the capacity of RAR for binding to the 5′regulatory region of ATG7 gene. Briefly, HCC cells were cross-linked in 1% formaldehyde/PBS for 10 min at 37 °C and were then washed twice with ice-cold PBS prior to be lysed. Chromatin fragments ranging from 200 to 1000 bp were obtained by sonication (SCIENTZ, China). The solution containing chromatin fragments was then incubated overnight at 4 °C with anti-RAR or anti-IgG. The chromatin–antibody complexes were then washed, eluted and reverse cross-linked at 65 °C for 6 h. The eluted DNA was purified with the phenol–chloroform. Immunoprecipitated chromatin was analyzed with PCR using primers targeting the human ATG7 promoter containing RAR binding motif. The primers used for ChIP-PCR are listed in Table 1.

### Evaluation of fluorescent LC3 puncta

The tandem mRFP-GFP-LC3 adenoviruses construct was obtained from Hanbio Inc (Shanghai, China) and was used to evaluate autophagy induction. Briefly, 5 × 10^4^ PLC/PRF/5 or HLE cells cultured on coverslips in 24- well microplates were transduced with mRFP-GFP-LC3 adenoviruses at 50 MOI. Two hours after transduction, removed the adenovirus by washing the cells with PBS, and then maintained the cells in complete culture medium with 40 μM ATRA or vehicle for 24 h. The cells were washed with PBS, fixed with 4% paraformaldehyde, and viewed under a fluorescence microscope. The number of mRFP, GFP and GFP-mRFP (yellow) dots were determined by manual counting of fluorescent puncta in five fields from three different cells. The number of dots per cell was obtained by dividing the total number of dots by the number of cell in each microscopic field.

### Determination of viability

Cell Counting Kit (CCK)-8 (CK04, Dojindo, Japan) was used to assess the cells viability. PLC/PRF/5 and HLE cells were seeded into 96-well microplates, and transfected with Si-Con or Si-ATG7. 24 h after transfection, the cells were subjected to 40 μM ATRA and solvent for another 24 h. The absorbance at 570 nm wavelength was measured with a Universal Microplate Reader (EL × 800). The percentage of cell viability was calculated as (A570_sample-background_)/ (A570_control-backgroud_).

### Caspase-3 activation and TUNEL assay

For caspase-3 activity assays, PLC/PRF/5 and HLE cells were homogenized in lysis buffer. Thereafter, 30µL lysates were added to a white 96-well plate, and then mixed with 60µL assay buffer. 90µL assay buffer was added into the blank well. After incubation for 10minutes at 37 °C, each well was added 10µL AC-DEVD-AFC at final concentration of 10 µg/mL, followed with further incubation for 1 h at 37 °C in the dark. The luminescence was measured using Imaging Multi-Mode Reader (BioTek). The changes of caspase-3 activity was calculated as [(A400_sample-blank_)/Protein concentration]/[(A400_control-blank_)/Protein concentration].

TUNEL assays of PLC/PRF/5 and HLE cells upon different treatment were carried out using a commercial cell death detection kit (Roche Applied Science, Germany) and analyzed as previously described^37^.

### Statistical analysis

The results of at least three separate experiments are presented as the mean ± s.d. Statistical significance was determined using the Two tailed Student’s *t* test, one-way and two-way ANOVA tests (SPSS 16 software).

## Supplementary Information

Supplementary Information.

Supplementary Information.
