# Supplementary material for: Intracellular alpha-fetoprotein interferes with all-trans retinoic acid induced ATG7 expression and autophagy in hepatocellular carcinoma cells
Source: Sci Rep. 2021 Jan 25;11:2146. doi: 10.1038/s41598-021-81678-7 (PMC7835378; doi:10.1038/s41598-021-81678-7)
Supplement: Supplementary file 1 — Supplementary Information. [file 41598_2021_81678_MOESM1_ESM.docx]

Supplementary Figure 1. Effect of ATRA on localization of RAR and autophagic flux in PLC/PRF/5 and HLE cells. A and B. Cells were treated with 40 μM ATRA for 4h and expression of total RAR and nuclear RAR were evaluated with Western blotting in PLC/PRF/5 (A, left panel) and HLE (A, right panel) cells. Densitometry quantification were performed, and ratio of RAR to corresponding loading control were calculated and marked. Expression and localization of RAR in PLC/PRF/5 (B, left panel) and HLE (B, right panel) cells following ATRA treatment were viewed and captured under laser confocal microscope. Nuclei and RAR were stained with DAPI (blue) and FITC (green), respectively. The image is representative of three independent experiments. C. Quantification of GFP/RFP puncta of Figure 1B. **P<0.01, compared with the solvent control. two-way ANOVA.

Supplementary Figure 2. Expression alteration of certain ATGs in HCC cells.

A, B and C. The expression at the mRNA level of ATGA7 (A), Beclin1 (B) and ATG5 (C) in PLC/PRF/5 and HepG2 cells upon ATRA treatment were determined with qRT-qPCR. D and E. Western blotting for the analyses of ATG5 in PLC/PRF/5 and HLE upon ATRA treatment. Densitometry quantification were performed, and ratio of ATG5 to loading control GAPDH were calculated and marked.

Supplementary Figure 3. Effect of autophagy blockade chloroquine on ATRA induced repression of cell viability. PLC/PRF/5 and HLE cells were treated with 40μM chloroquine alone or in combination with 40μM ATRA for 24h. A and B. Western blotting analyses for p62/SQSTM1 and LC3 conversion. Densitometry quantification were performed, and ratio of p62/SQSTM1 to GAPDH, and LC3II to LC3I were calculated and marked. C and D. Cell viability of PLC/PRF/5 and HLE cells with CCK-8 method. * P< 0.05, and ** P< 0.01, compared with Control group. one-way ANOVA.

Supplementary Figure 4. Effect of AFP depletion or ectopic expression on ATRA induced expression of ATG7 and autophagy in HCC cells. PLC/PRF/5 cells were transfected with shRNA923 for depletion of AFP, and HLE cells were transiently transfected with pcDNA3.1 (+)-AFP for ectopic expressive of AFP, which were followed with ATRA treatment for 24h. Expression of AFP, ATG7, p62/SQSTM1 and LC3 were analyzed with Western blotting in PLC/PRF/5 (A) and HLE (B) cells. GAPDH was used as loading control. Densitometry of the blots were quantified, and the ratio of AFP, ATG7, and p62/SQSTM1 to GAPDH, or LC3II to LC3I were calculated and marked.

Supplementary Figure 5. Schematic overview of role for intracellular AFP in ATRA induced ATG7 expression, autophagy and apoptosis in HCC cells.
