# Supplementary material for: Intracellular alpha-fetoprotein interferes with all-trans retinoic acid induced ATG7 expression and autophagy in hepatocellular carcinoma cells
Source: Sci Rep. 2021 Jan 25;11:2146. doi: 10.1038/s41598-021-81678-7 (PMC7835378; doi:10.1038/s41598-021-81678-7)
Supplement: Supplementary file 2 — Supplementary Information. [file 41598_2021_81678_MOESM2_ESM.pdf]

Intracellular Alpha-fetoprotein Interferes with All-trans retinoic acid induced ATG7 expression and Autophagy in Hepatocellular carcinoma cells

Shanshan Wang[1], Rilu Feng[2], Ying Shi[1], Dexi Chen[1], Honglei Weng[2], Huiguo Ding[3], Chenguang Zhang[4]*

[1] Beijing Institute of Hepatology, Beijing You' An Hospital, Capital Medical University, Beijing, 100069, China

[2] Department of Medicine II, Medical Faculty Mannheim, Heidelberg University, Mannheim, 68167, Germany

[3]Department of Gastroenterology and Hepatology, Beijing You'an Hospital, Capital Medical University, Beijing 100069, China

[4]Department of Biochemistry and Molecular Biology, school of basic medical sciences, Capital Medical University, Beijing 100069, China

* Corresponding author: chzhang@ccmu.edu.cn

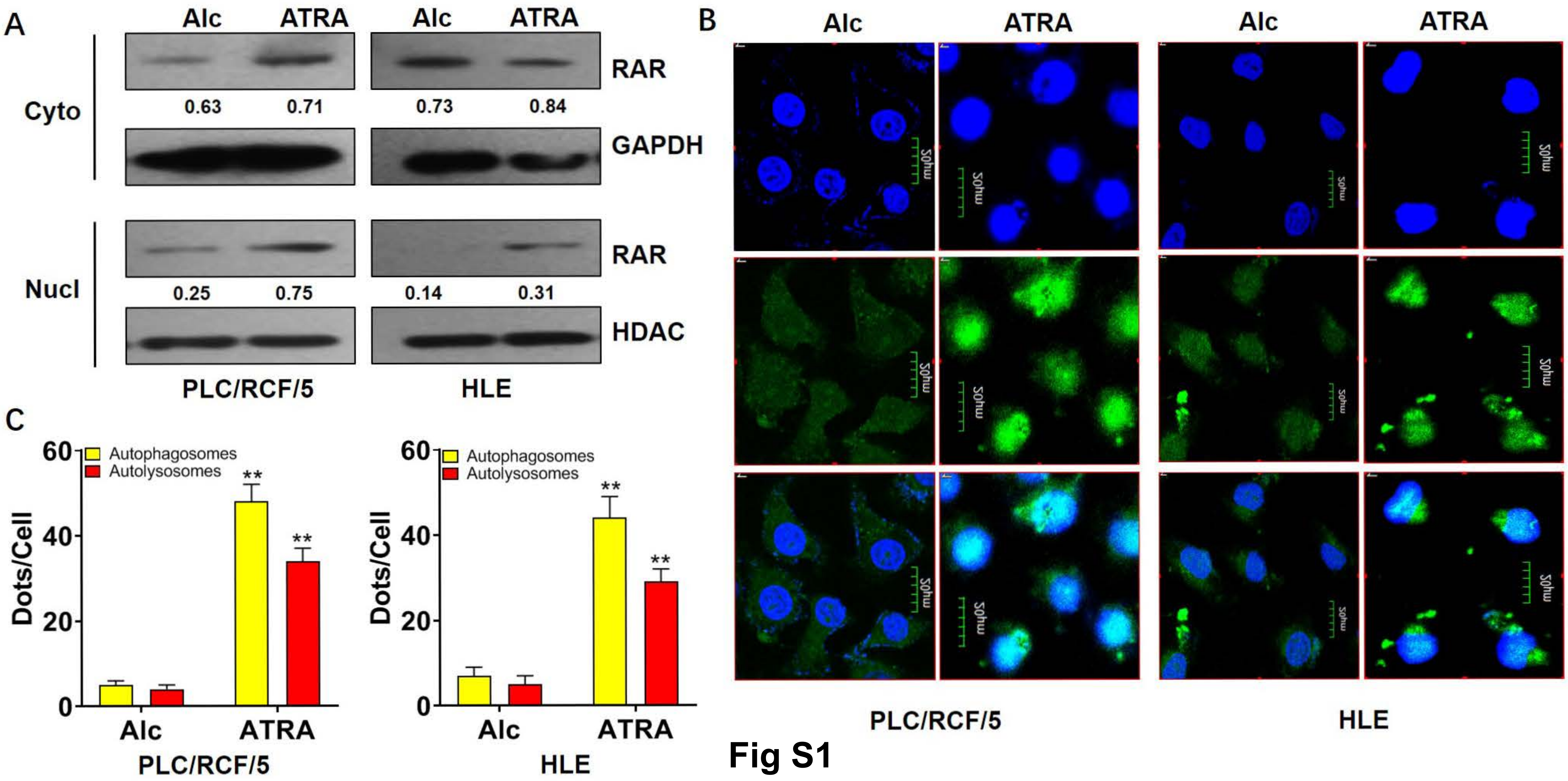
A
Alc
ATRA
Alc
ATRA
Cyto
RAR
0.63
0.71
0.73
0.84
GAPDH
Nucl
RAR
0.25
0.75
0.14
0.31
HDAC
PLC/RCF/5
HLE
B
Alc
ATRA
Alc
ATRA
PLC/RCF/5
HLE
C
Autophagosomes
Autolysosomes
Dots/Cell
60
40
20
0
**
**
Alc
ATRA
PLC/RCF/5
Autophagosomes
Autolysosomes
Dots/Cell
60
40
20
0
**
**
Alc
ATRA
HLE

Fig S1

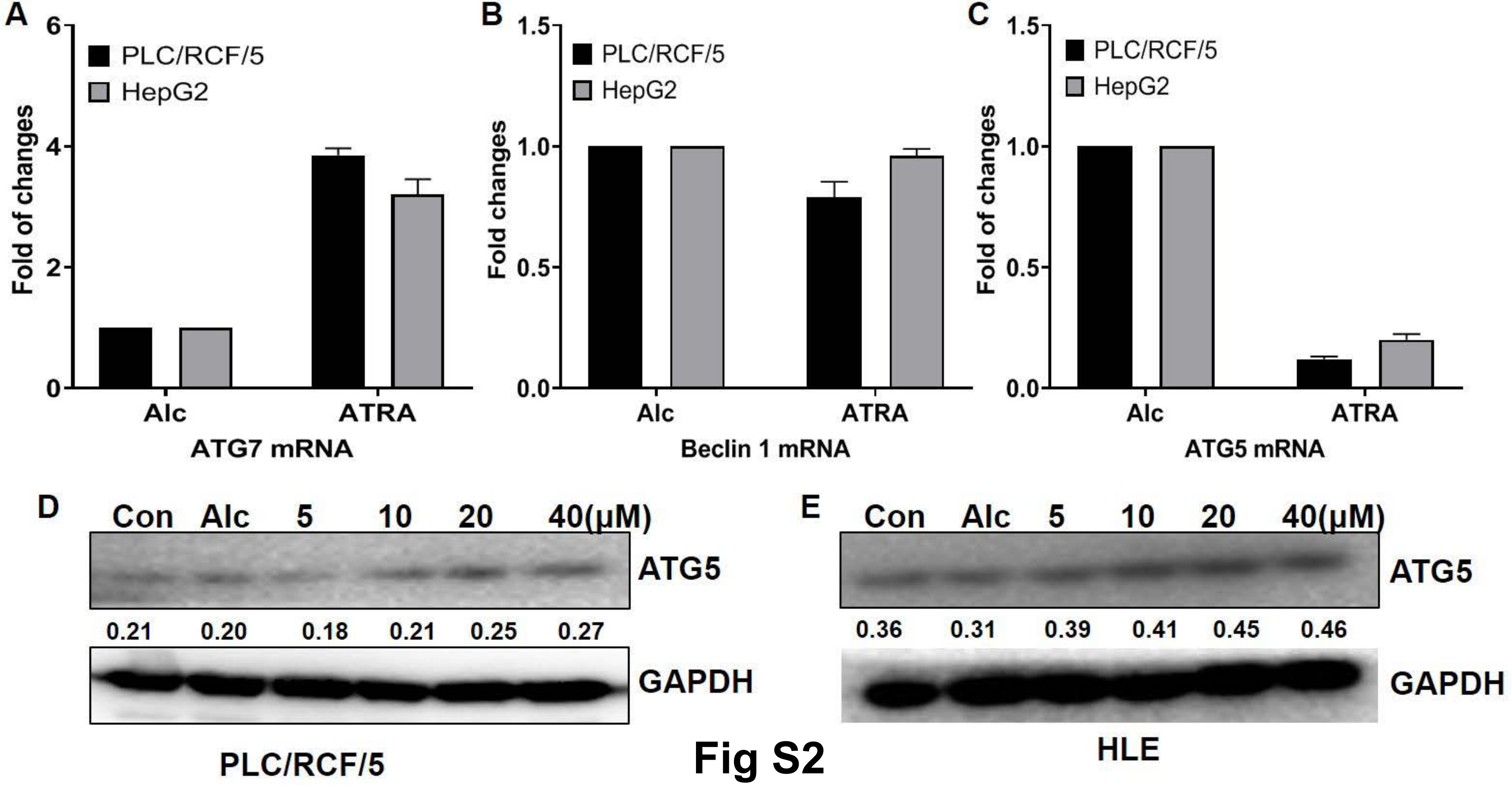

A
B
C
PLC/RCF/5
HepG2
Fold of changes
Fold changes
6
4
2
0
1.5
1.0
0.5
0.0
Alc
ATRA
ATG7 mRNA
Beclin 1 mRNA
ATG5 mRNA
D
E
Con
Alc
5
10
20
40(μM)
ATG5
GAPDH
0.21
0.20
0.18
0.21
0.25
0.27
0.36
0.31
0.39
0.41
0.45
0.46
PLC/RCF/5
HLE

Fig S2

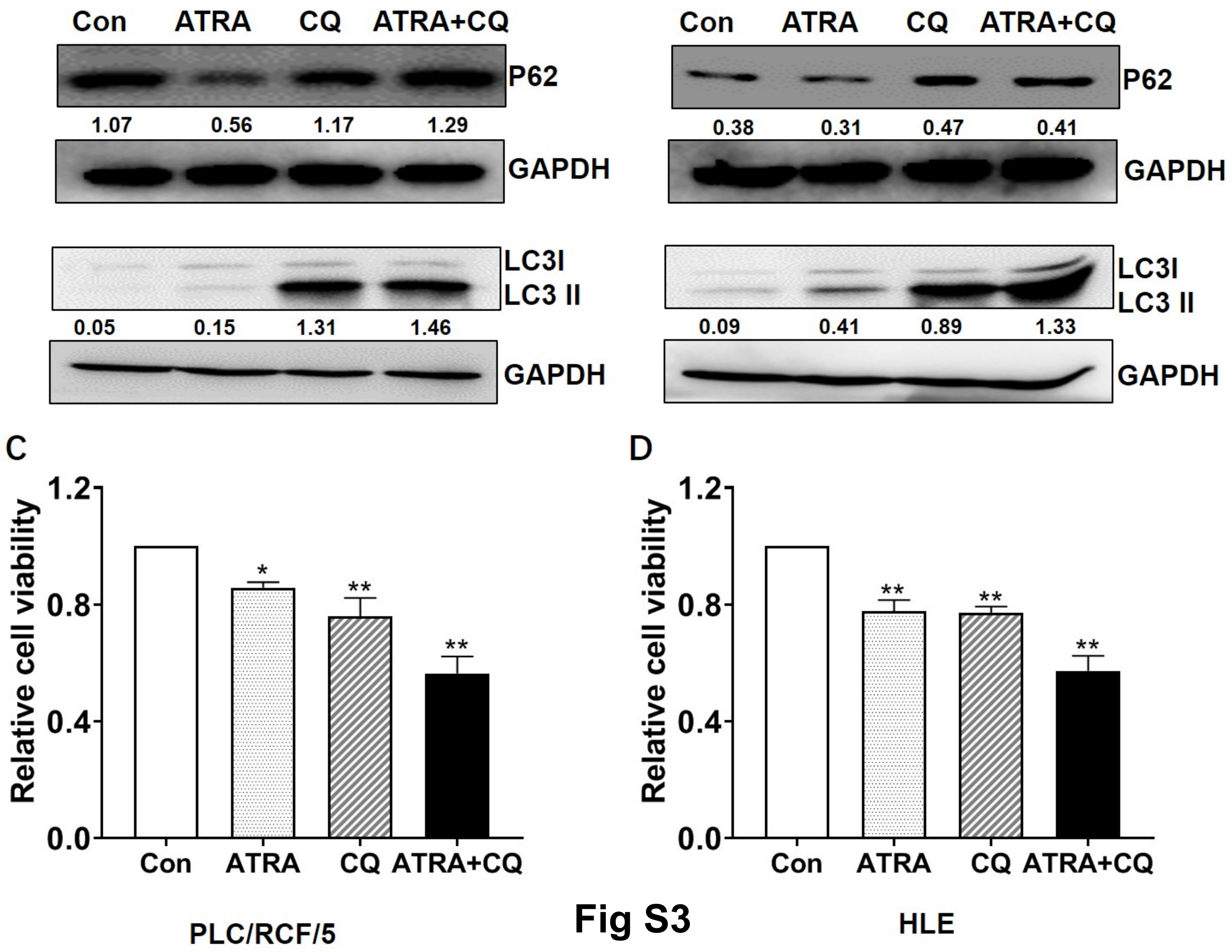
Con
ATRA
CQ
ATRA+CQ
P62
1.07
0.56
1.17
1.29
GAPDH
LC3I
LC3 II
0.05
0.15
1.31
1.46
GAPDH
Con
ATRA
CQ
ATRA+CQ
P62
0.38
0.31
0.47
0.41
GAPDH
LC3I
LC3 II
0.09
0.41
0.89
1.33
GAPDH
C
Relative cell viability
1.2
0.8
0.4
0.0
*
**
**
Con
ATRA
CQ
ATRA+CQ
PLC/RCF/5
D
Relative cell viability
1.2
0.8
0.4
0.0
**
**
**
Con
ATRA
CQ
ATRA+CQ
HLE

Fig S3

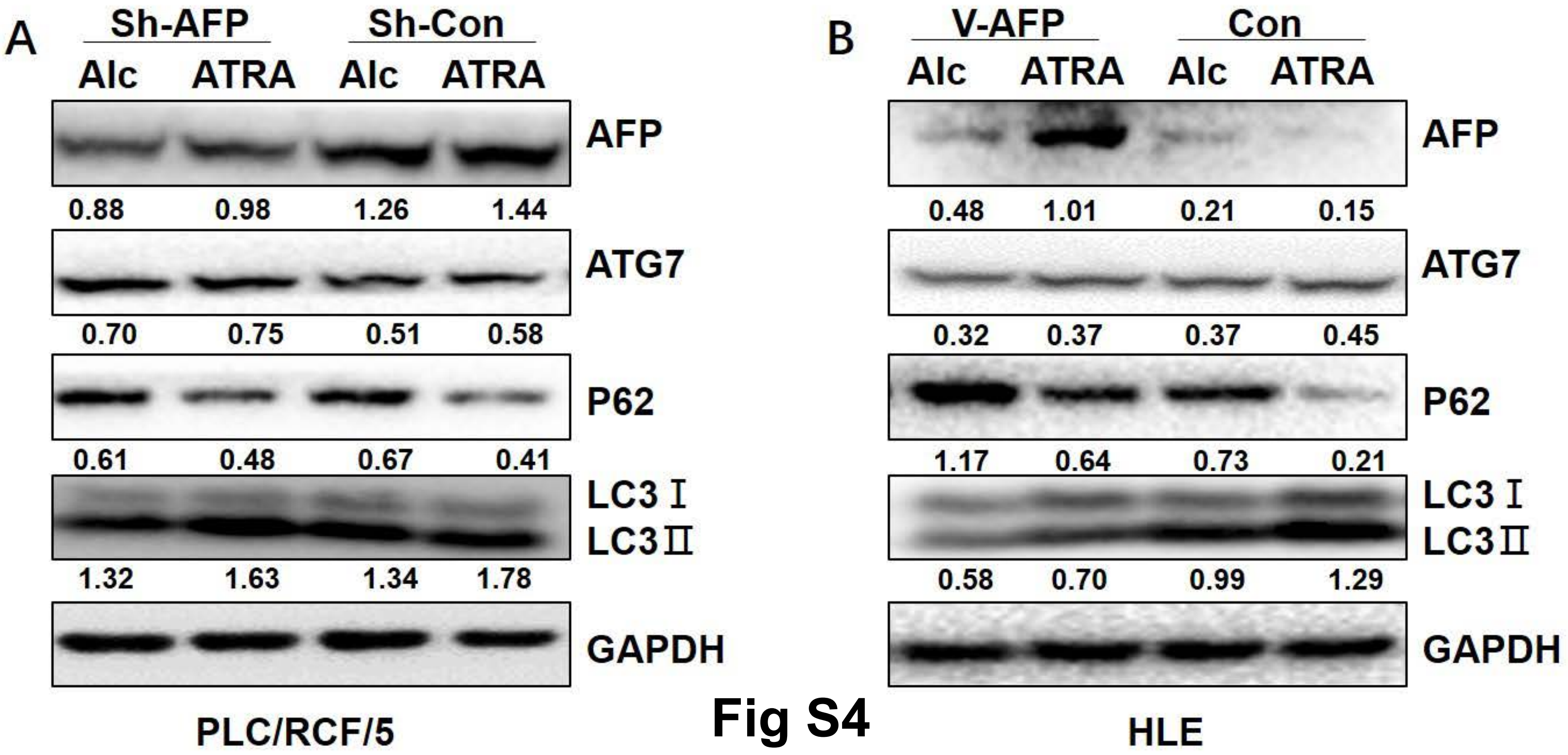
A
Sh-AFP
Sh-Con
Alc
ATRA
Alc
ATRA
AFP
0.88
0.98
1.26
1.44
ATG7
0.70
0.75
0.51
0.58
P62
0.61
0.48
0.67
0.41
LC3 I
LC3 II
1.32
1.63
1.34
1.78
GAPDH
PLC/RCF/5
B
V-AFP
Con
Alc
ATRA
Alc
ATRA
AFP
0.48
1.01
0.21
0.15
ATG7
0.32
0.37
0.37
0.45
P62
1.17
0.64
0.73
0.21
LC3 I
LC3 II
0.58
0.70
0.99
1.29
GAPDH
HLE

Fig S4

Fig S5

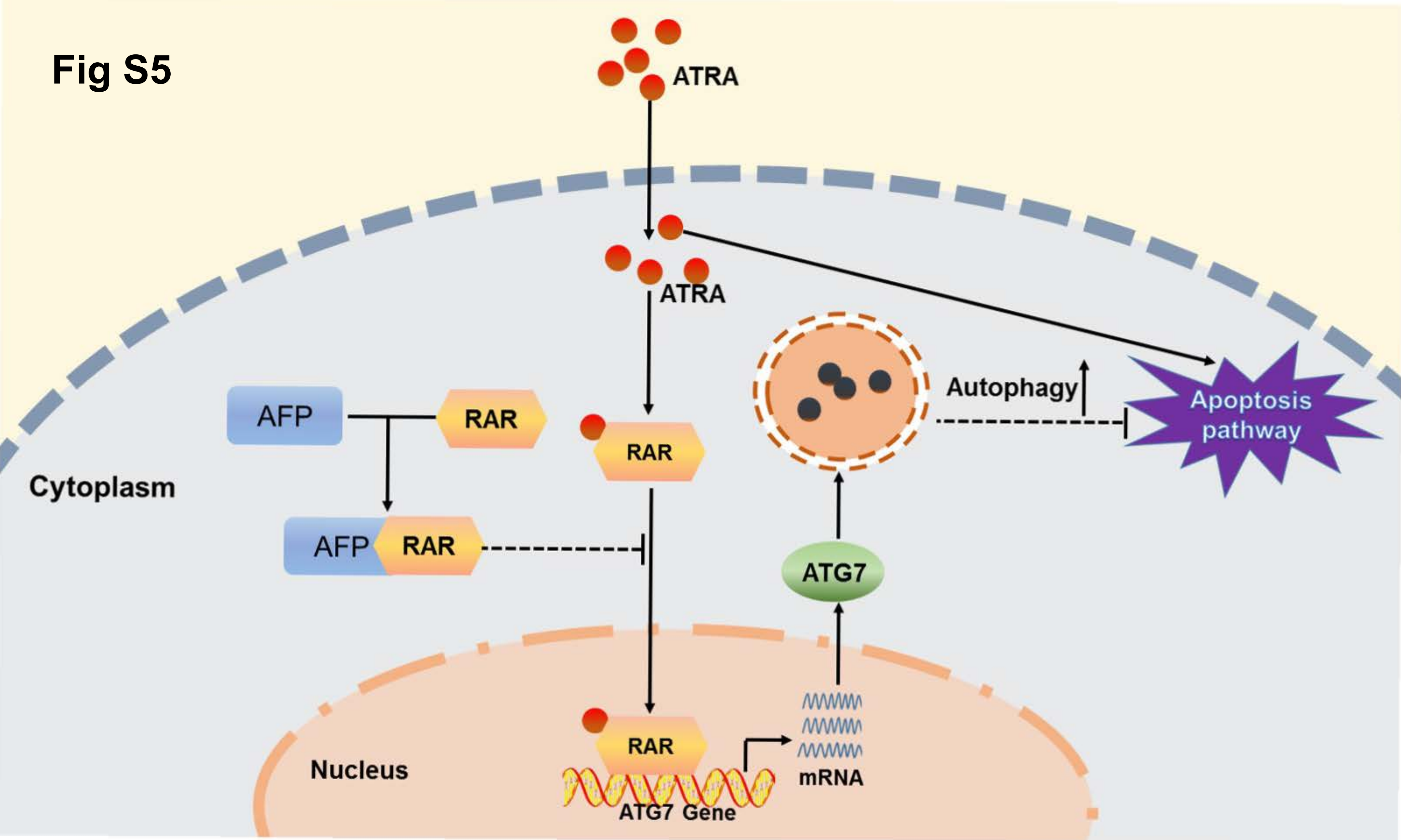

ATRA
ATRA
AFP
RAR
RAR
AFP
RAR
Cytoplasm
Autophagy
Apoptosis pathway
ATG7
RAR
Nucleus
mRNA
ATG7 Gene

Fig 1A

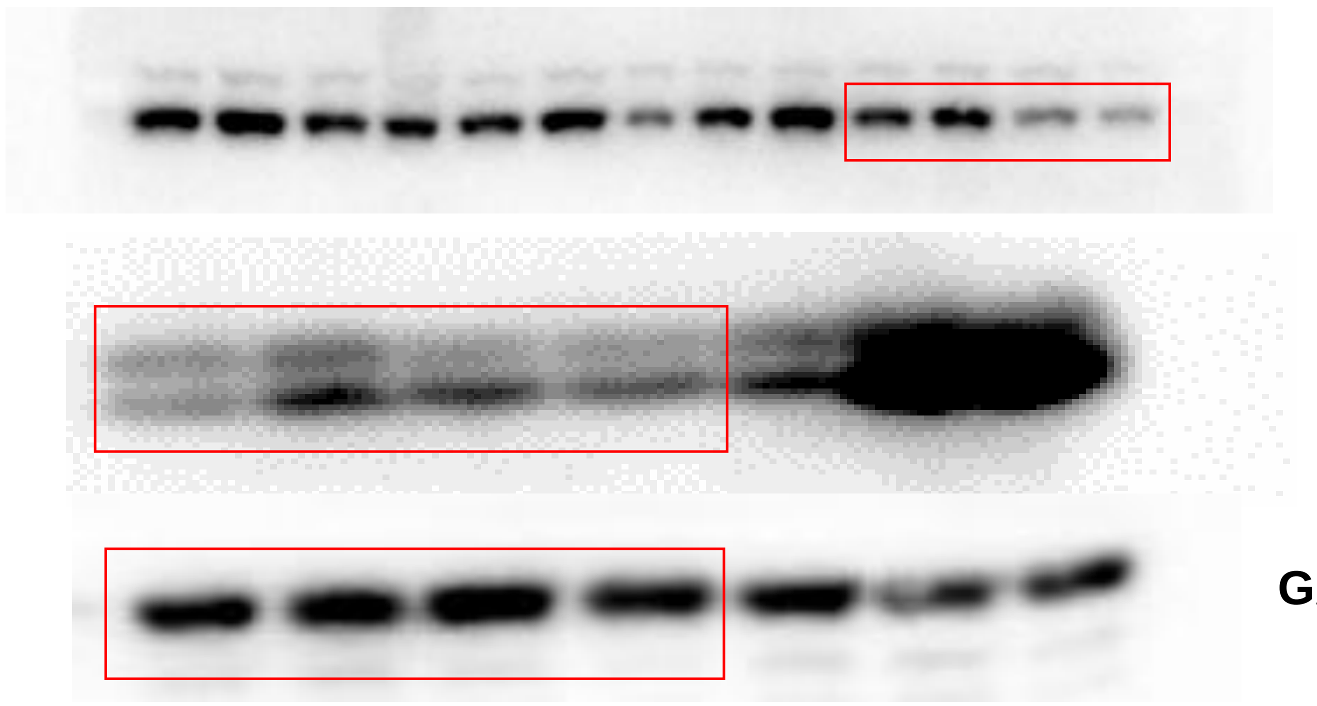

Fig 1A‘

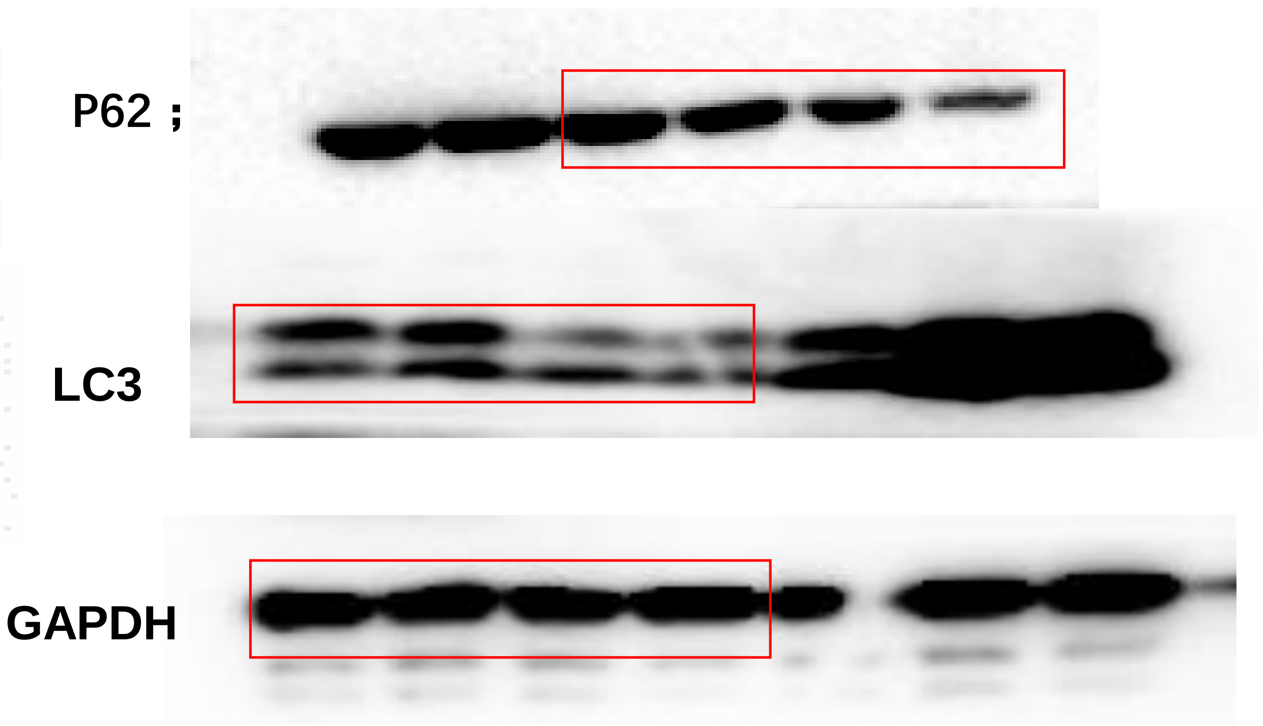

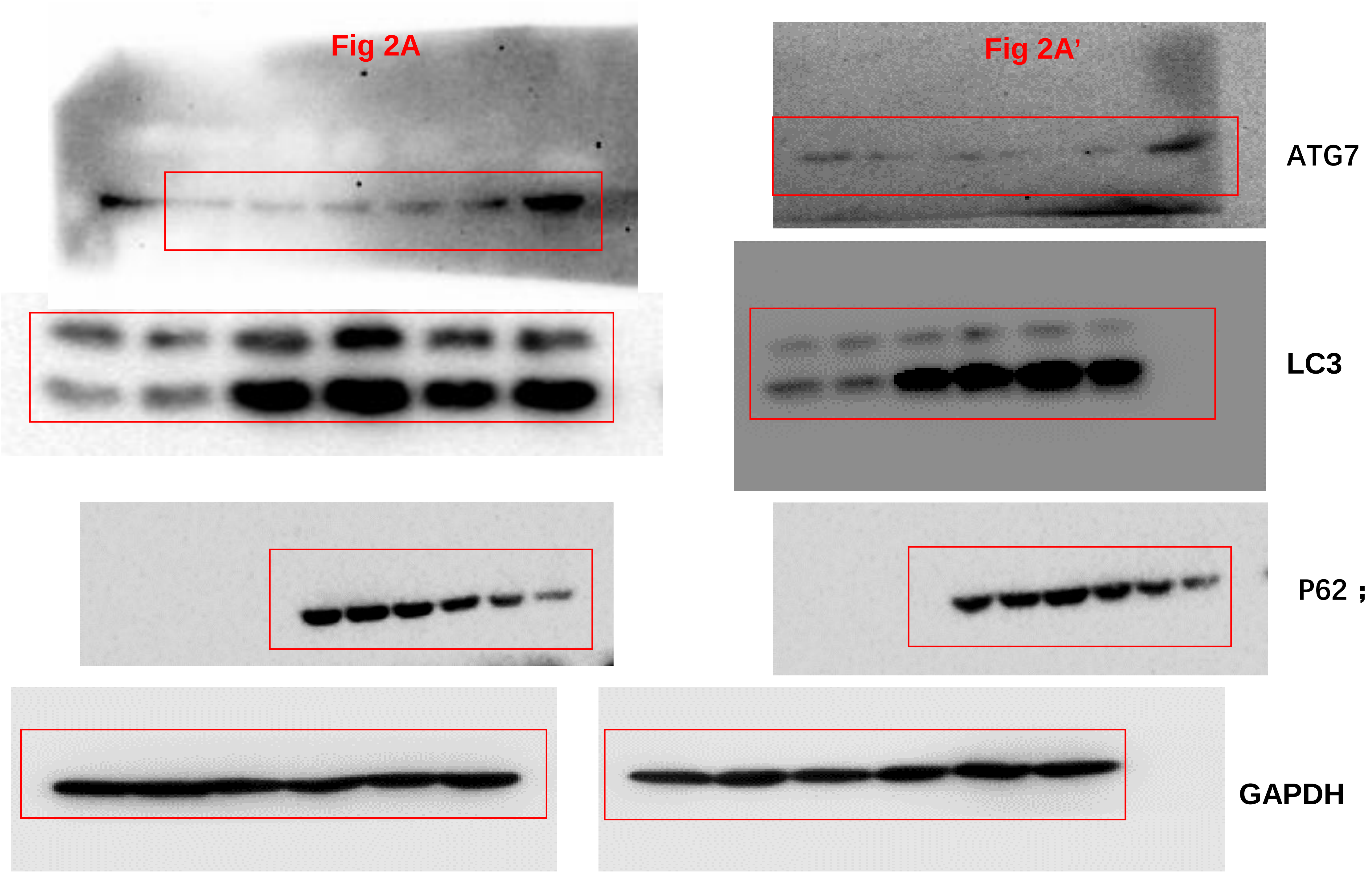

Fig 2A
Fig 2A'
ATG7
LC3
P62 ;
GAPDH

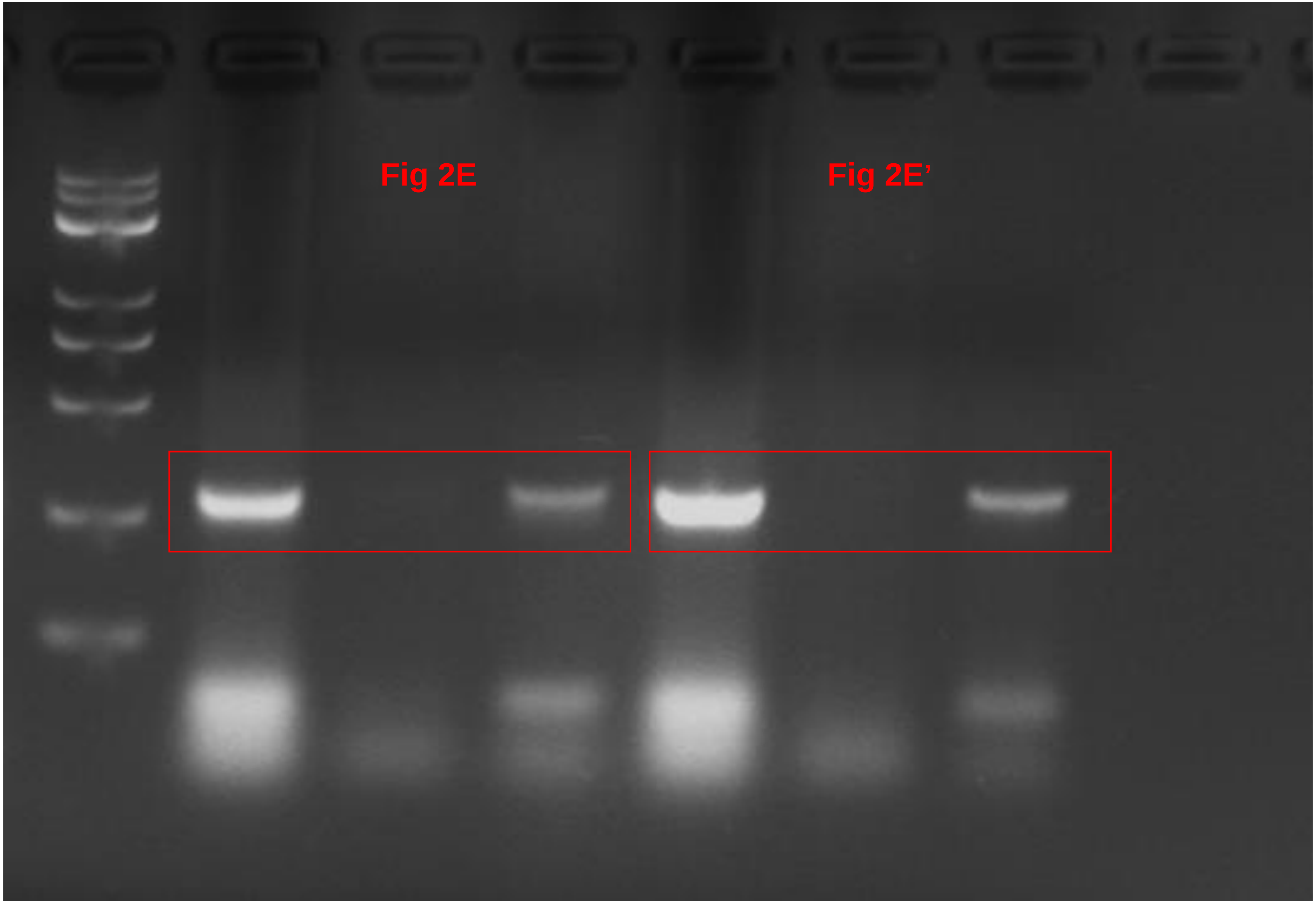
Fig 2E
Fig 2E'

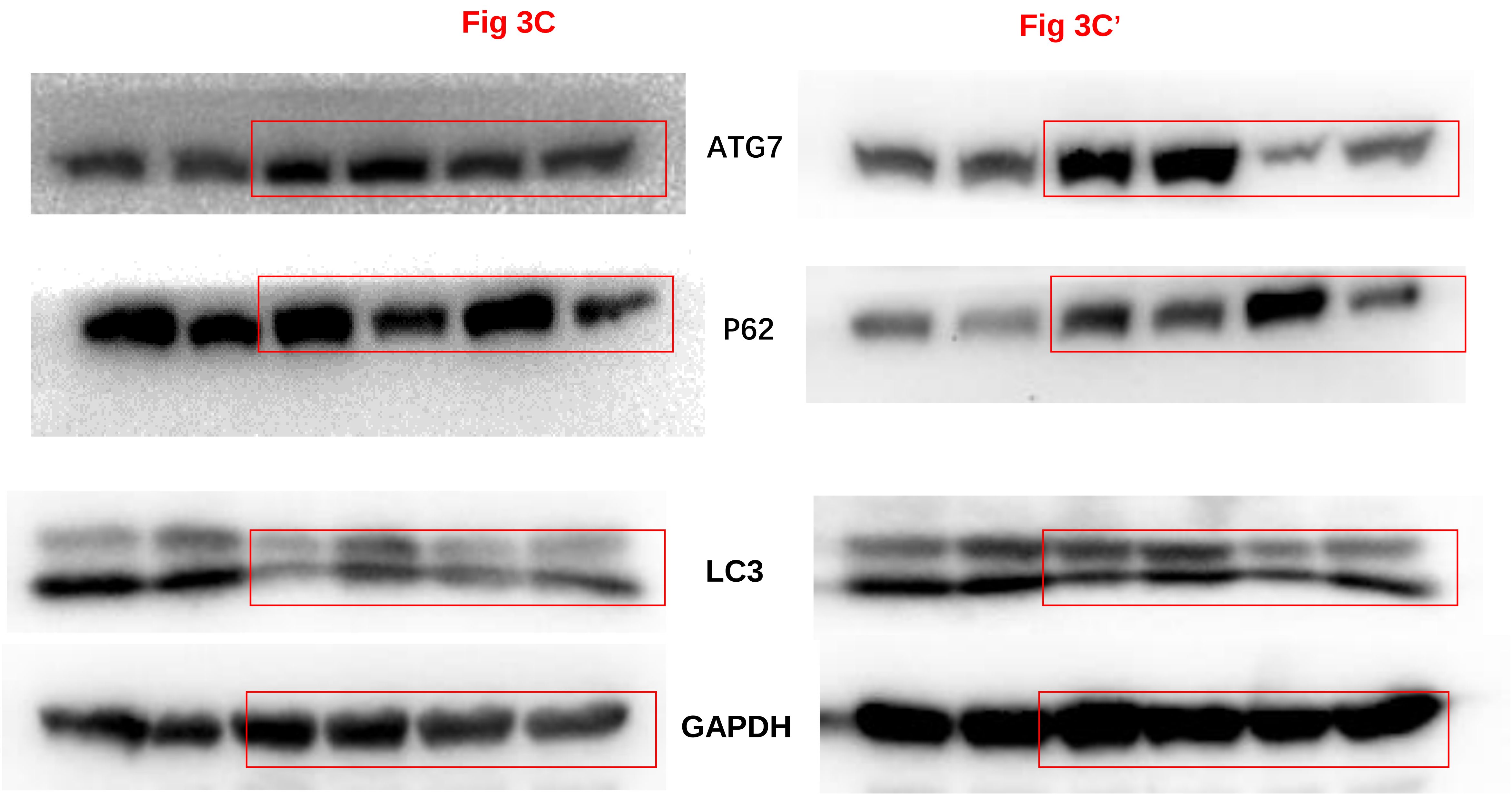
Fig 3C
Fig 3C'
ATG7
P62
LC3
GAPDH

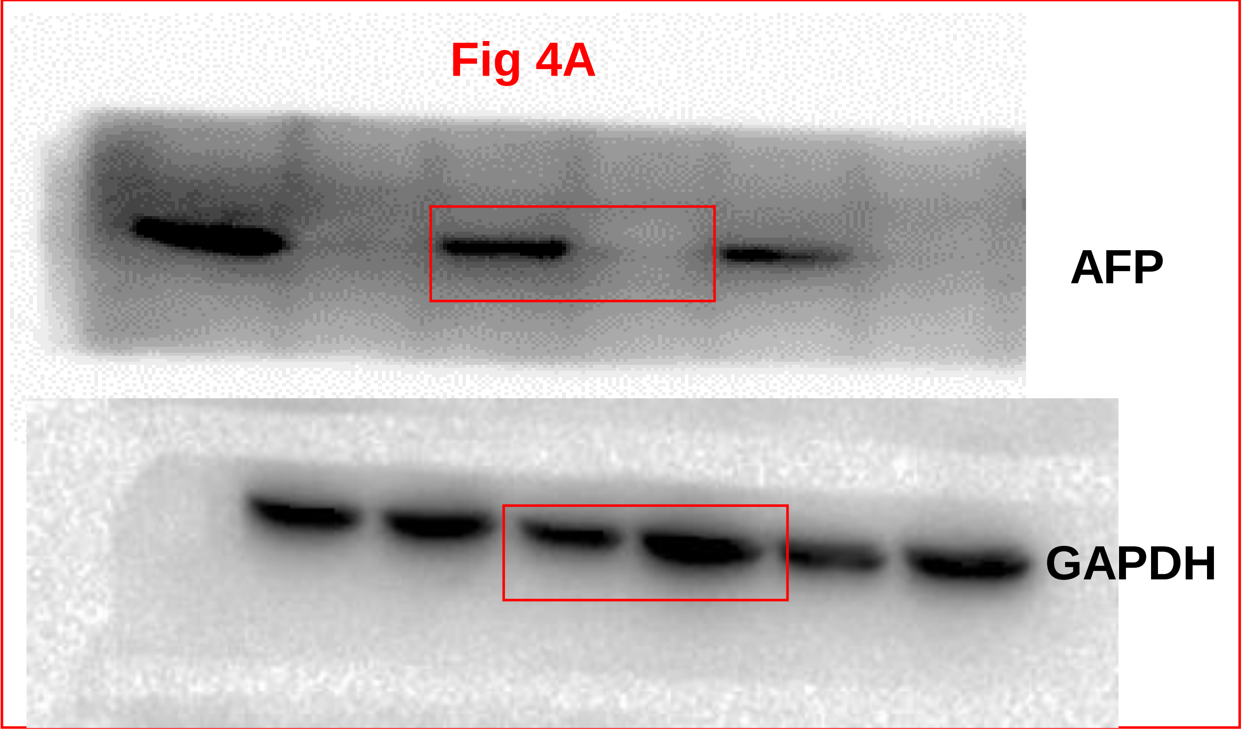
Fig 4A
AFP
GAPDH

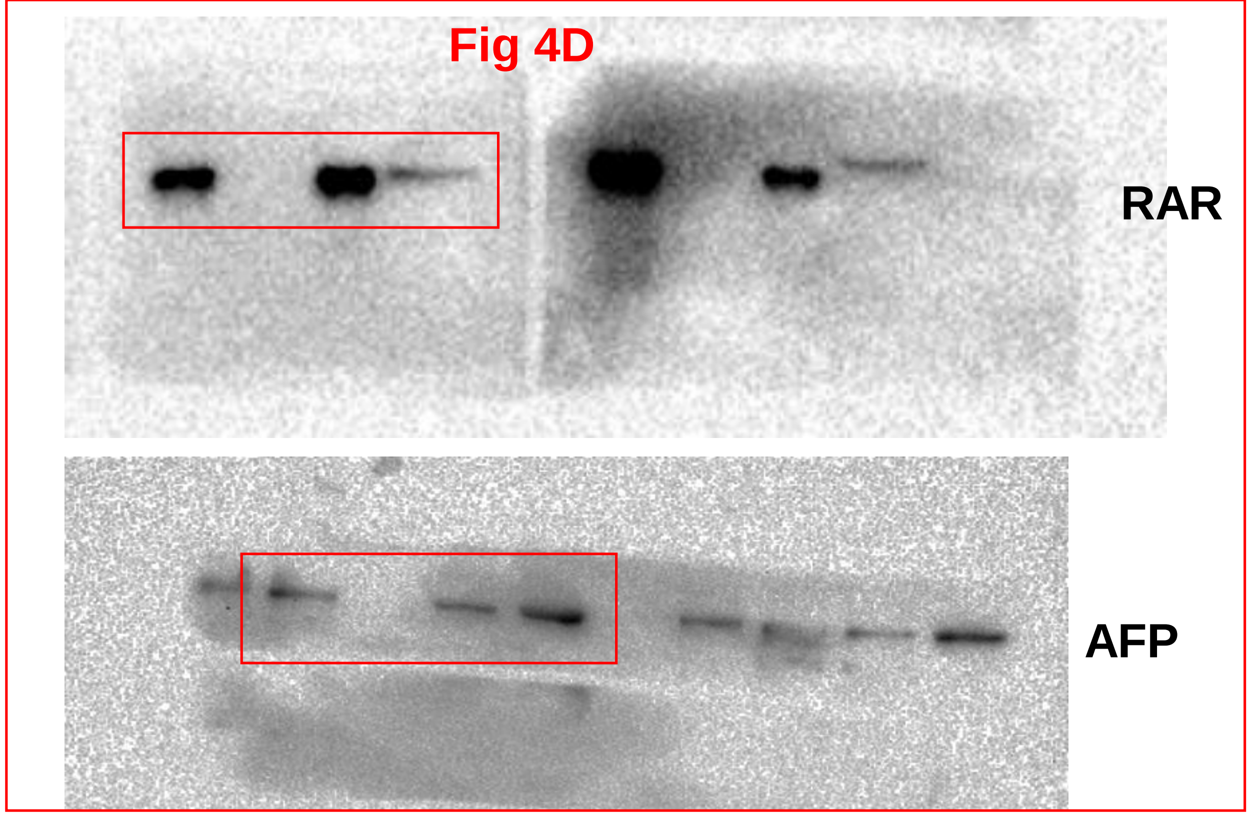
Fig 4D
RAR
AFP

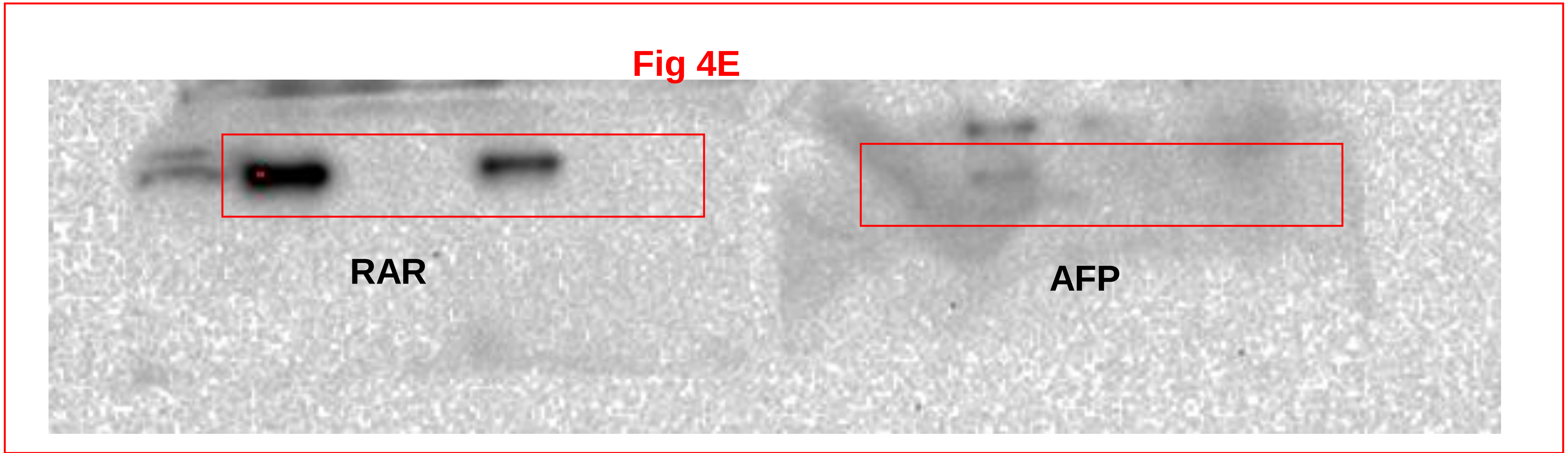
Fig 4E
RAR
AFP

Fig 5B

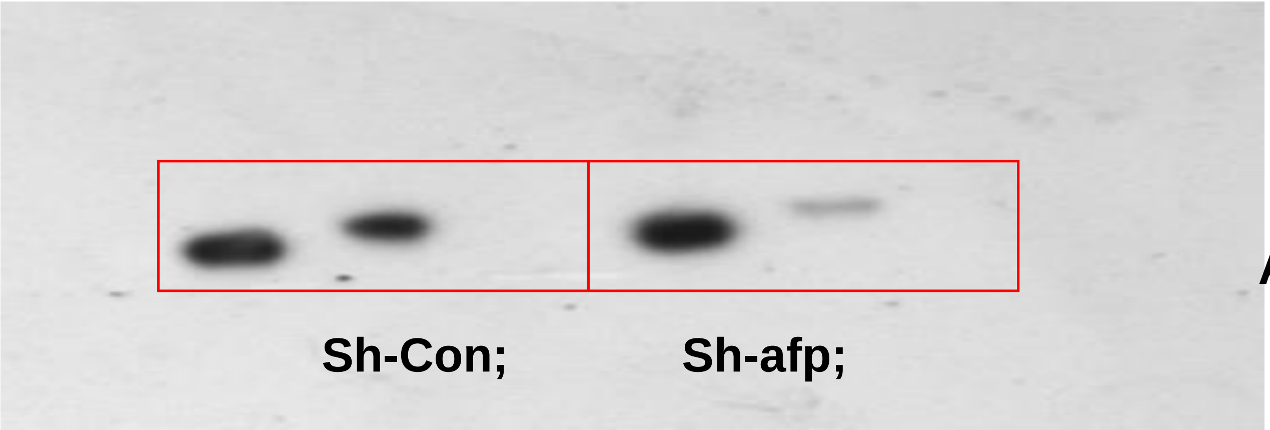

AFP

Fig 5B'

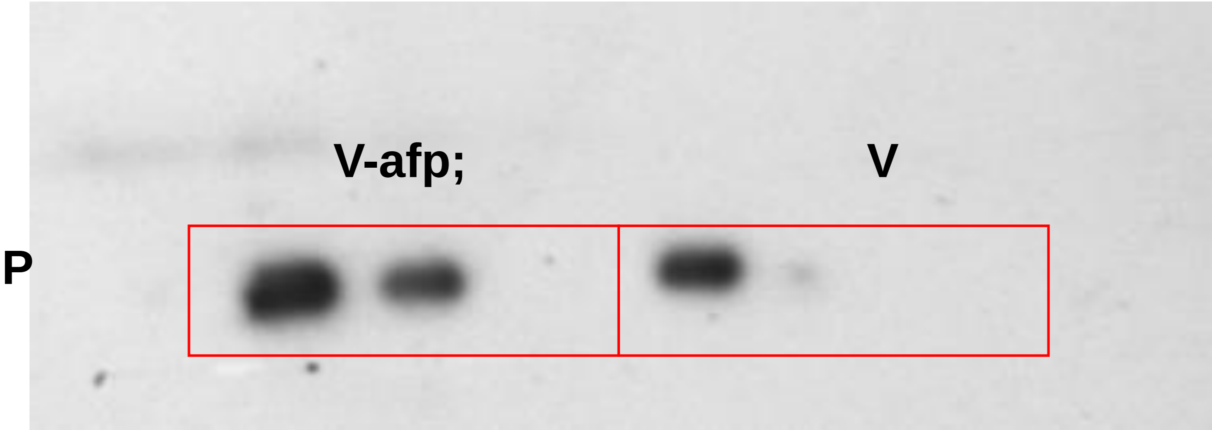

Fig 5C

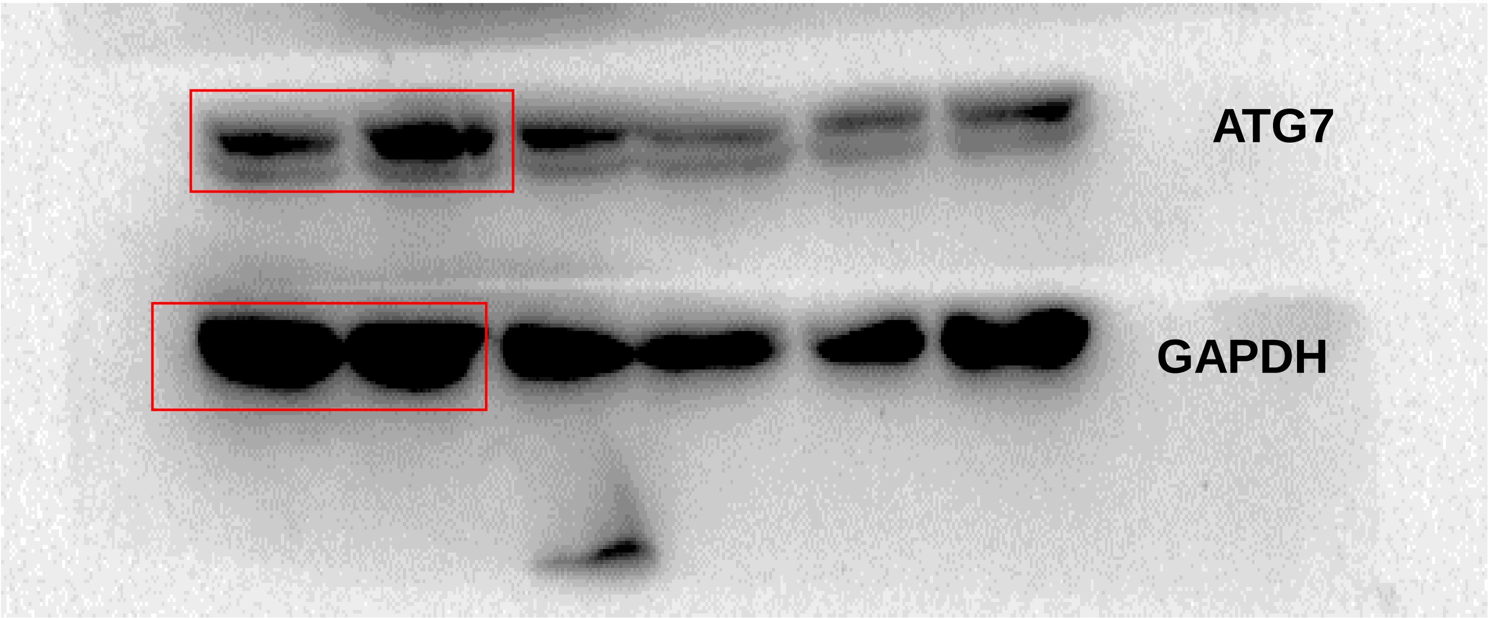

Fig 5C'

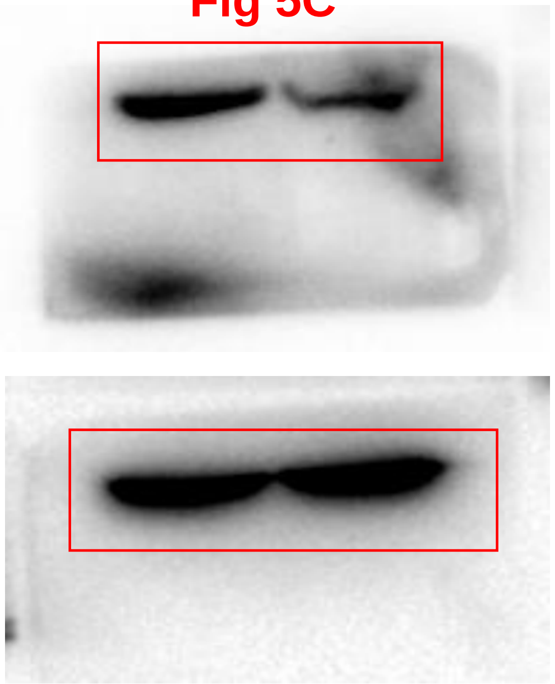

ATG7

GAPDH

Fig S1A

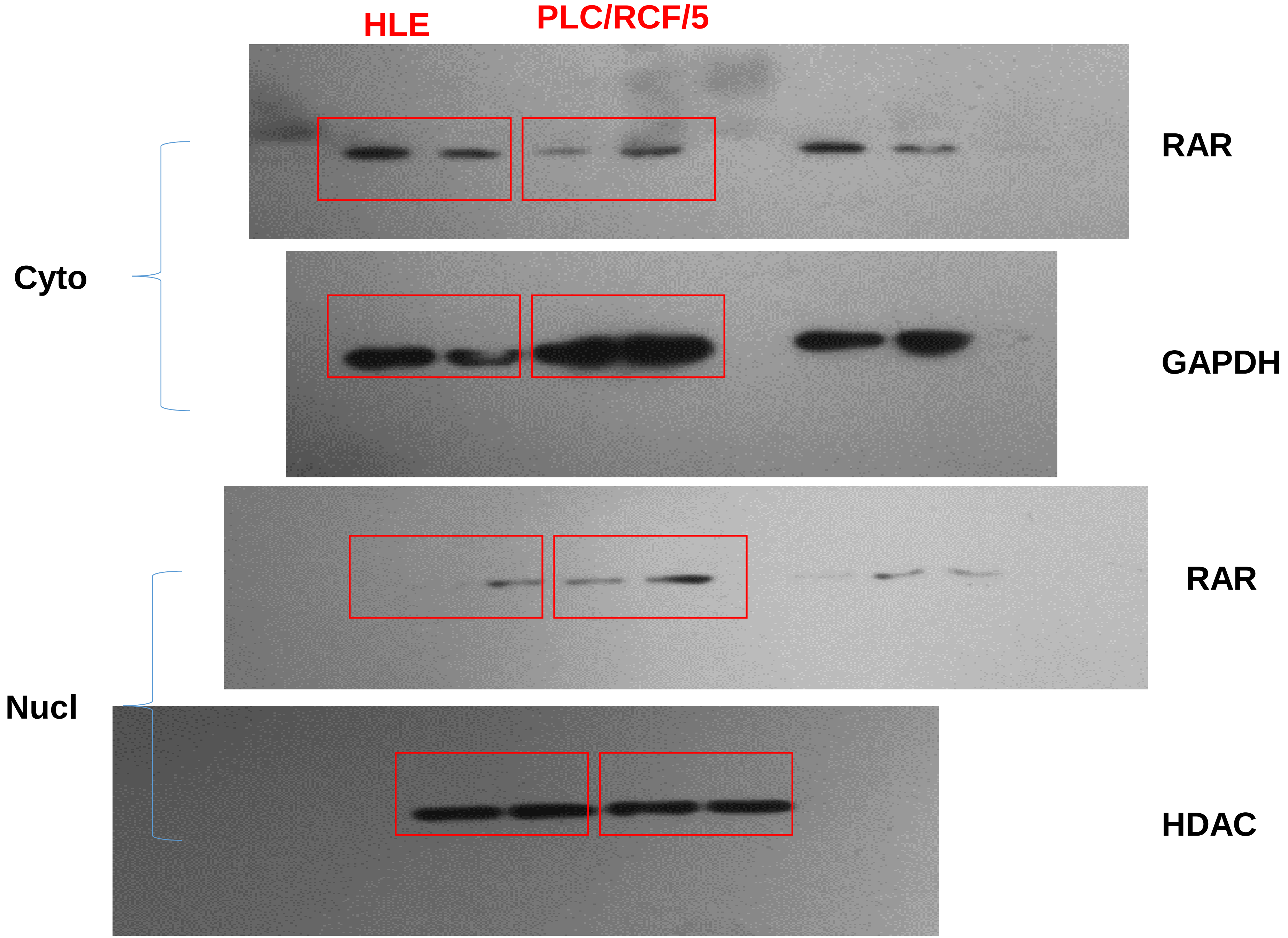

Fig S2D

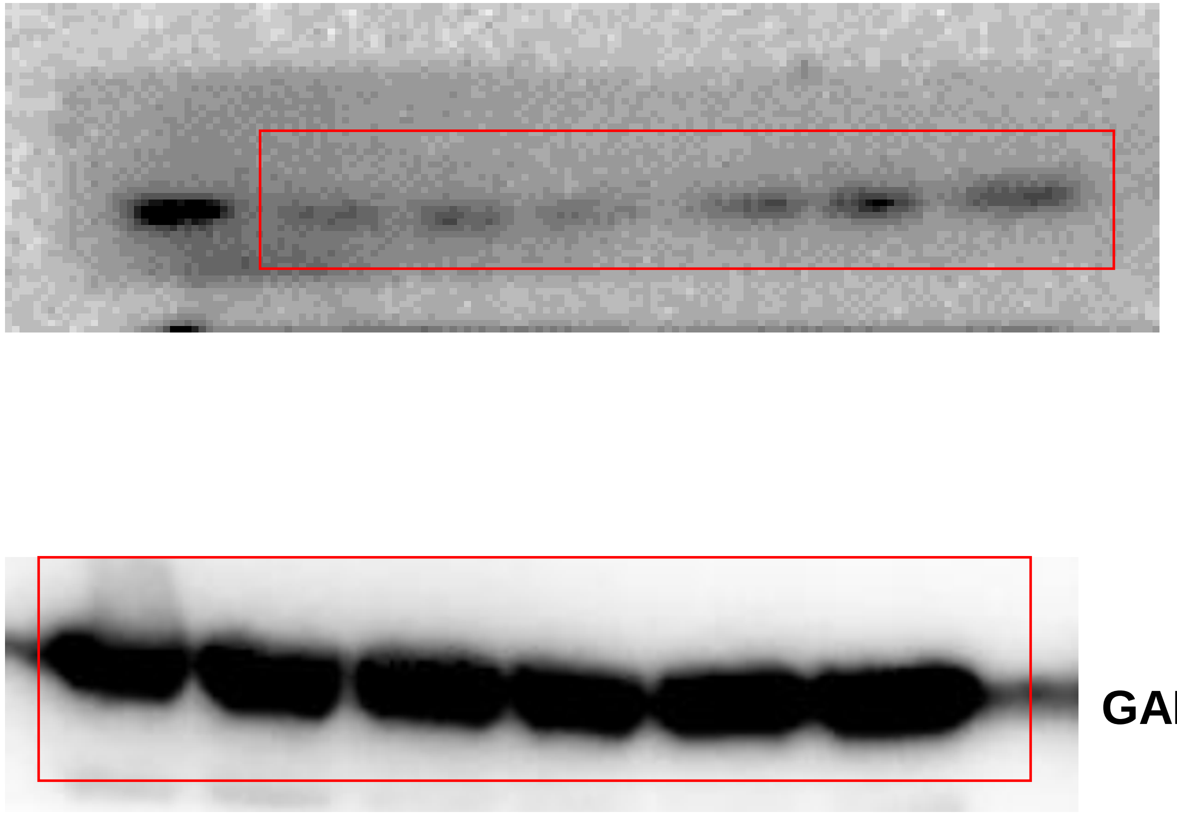

Fig S2E

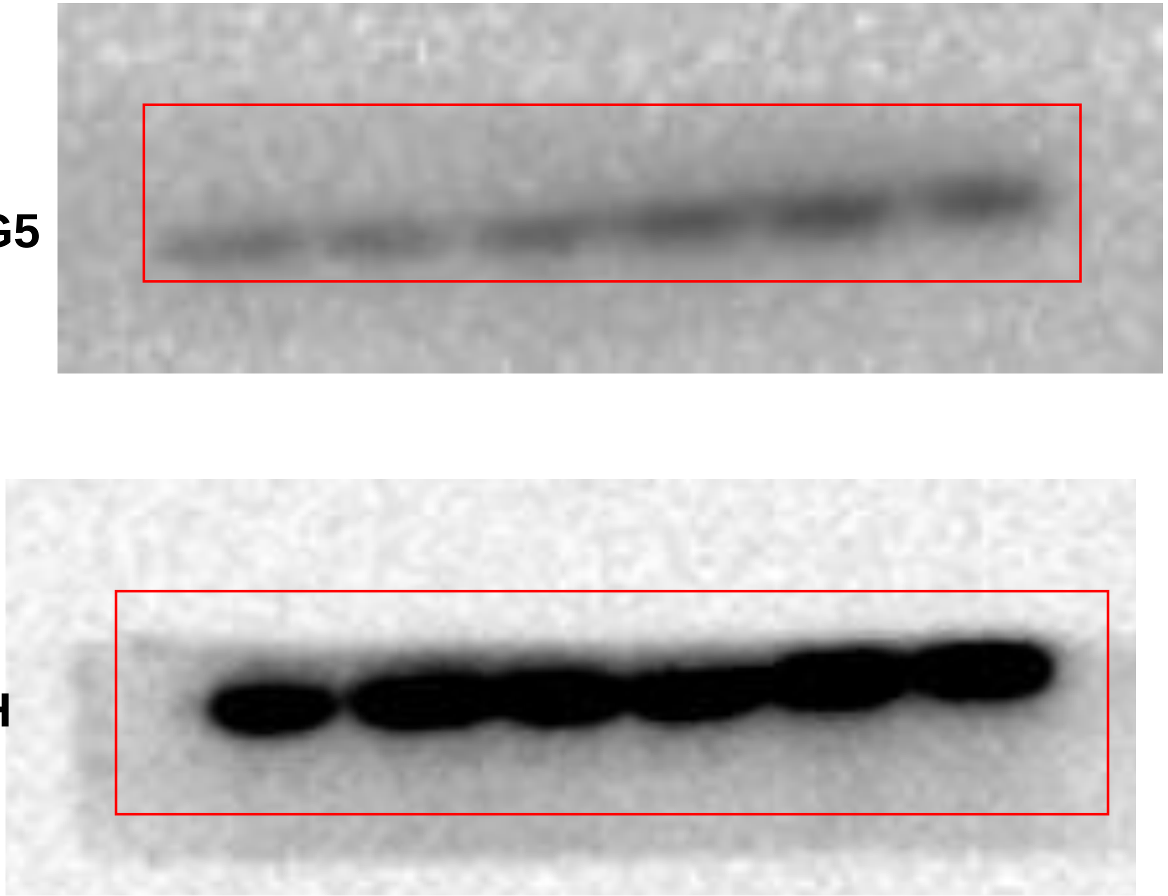

ATG5

GAPDH

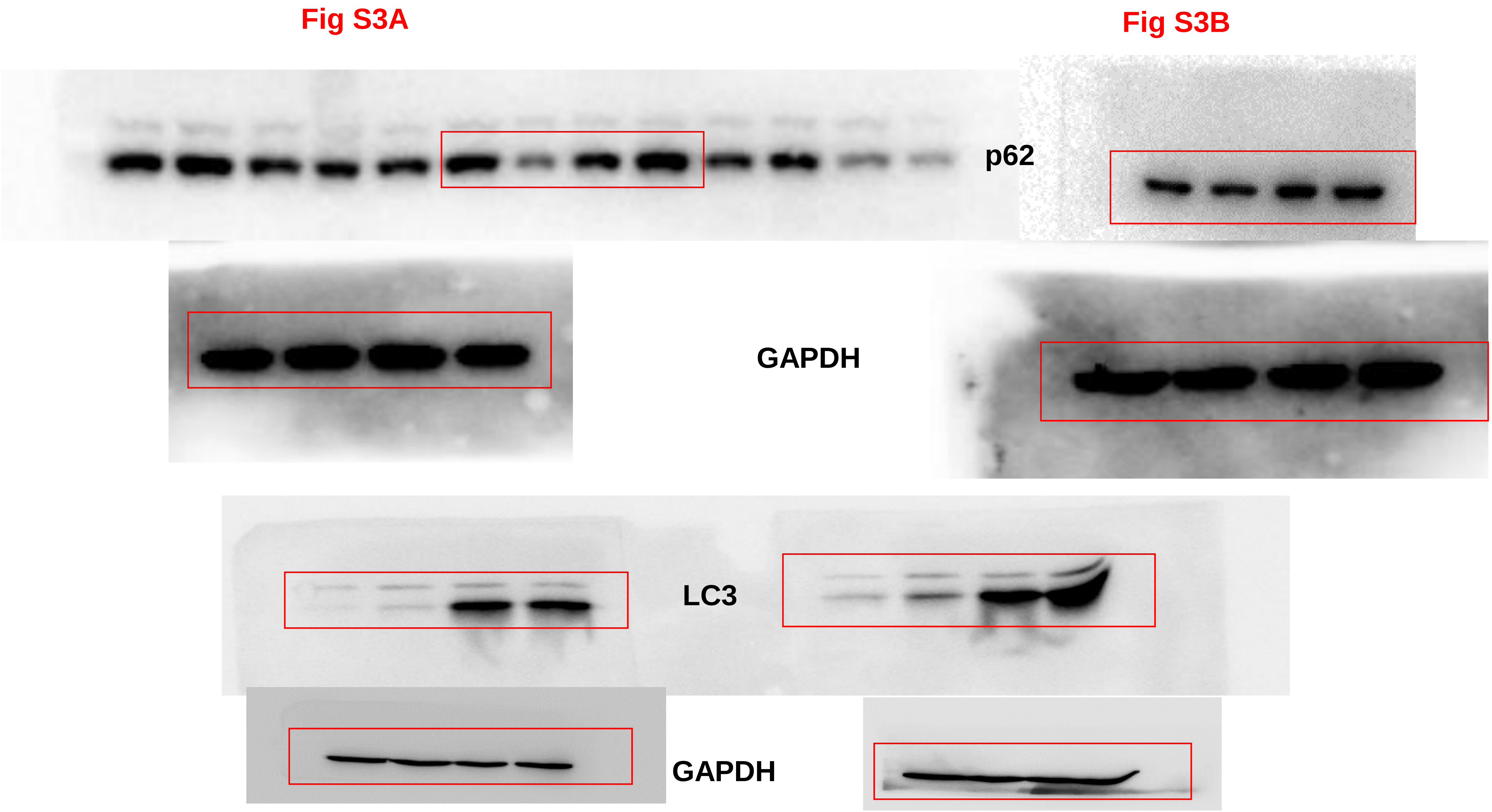
Fig S3A
Fig S3B
p62
GAPDH
LC3
GAPDH

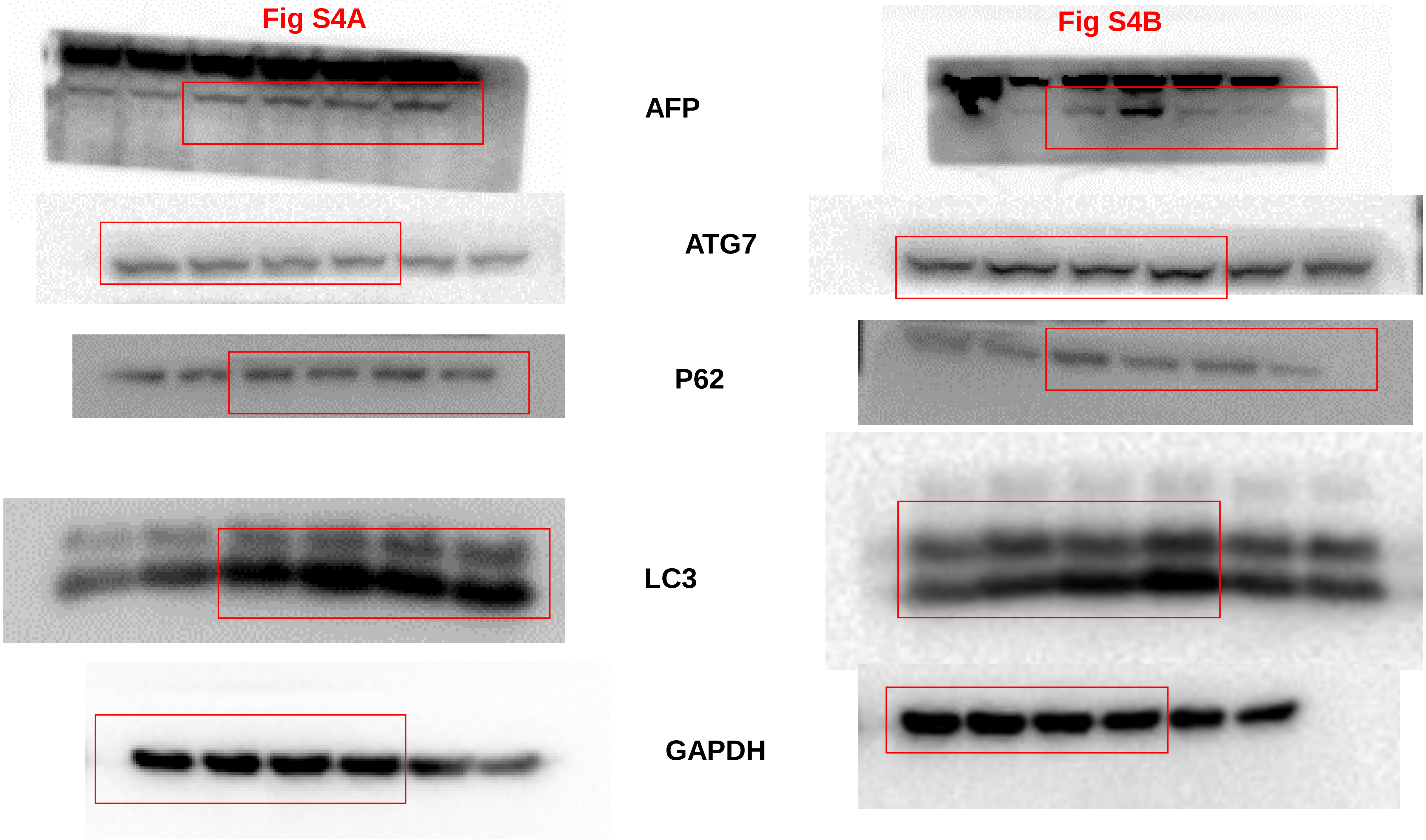
Fig S4A
Fig S4B
AFP
ATG7
P62
LC3
GAPDH
